# Supplementary material for: pTINCR microprotein promotes epithelial differentiation and suppresses tumor growth through CDC42 SUMOylation and activation
Source: Nat Commun. 2022 Nov 11;13:6840. doi: 10.1038/s41467-022-34529-6 (PMC9652315; doi:10.1038/s41467-022-34529-6)

pTINCR microprotein promotes epithelial differentiation and suppresses tumor growth through CDC42 SUMOylation and activation

Boix *et al.*

Supplementary Information

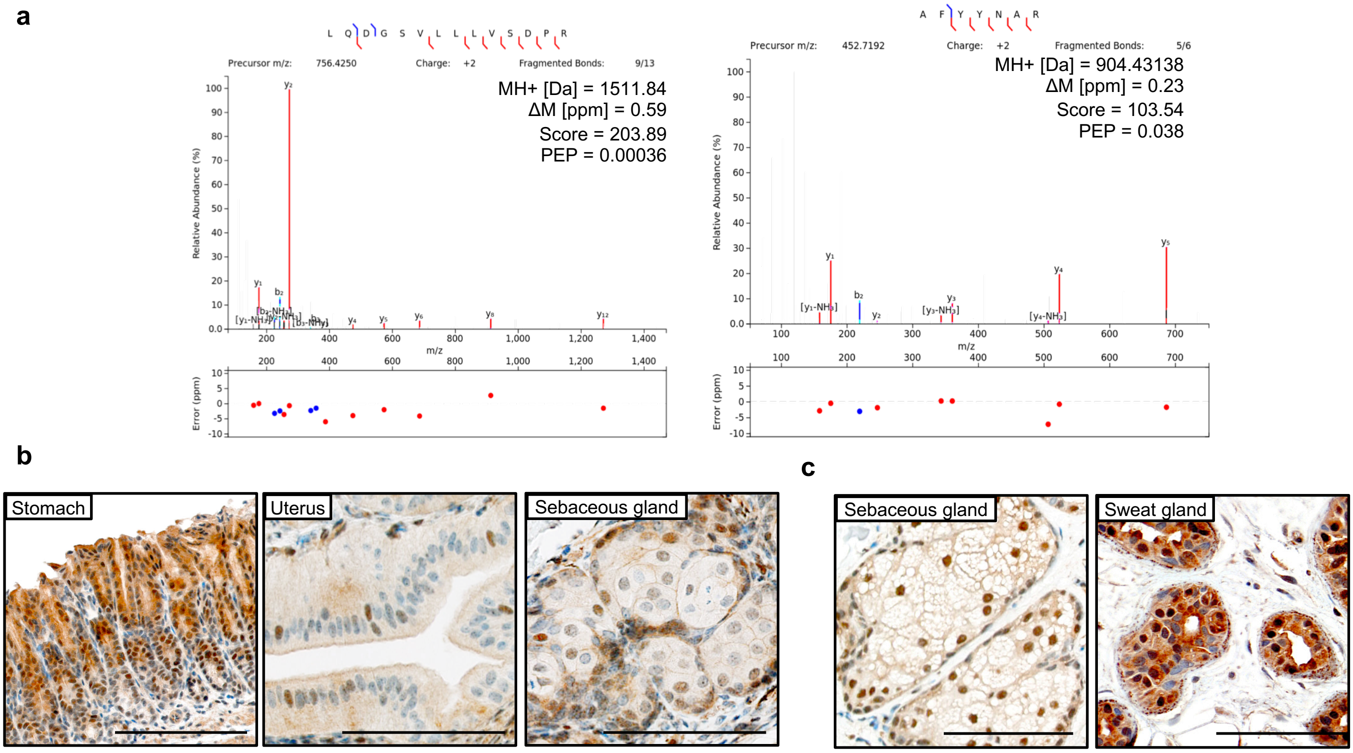

Supplementary Fig. 1 | Detection of endogenous pTINCR in mouse and human tissues.
a. MS/MS spectra of two unique peptides derived from pTINCR microprotein identified by mass spectrometry in human skin organotypic cultures.
b and c. Representative immunostaining images of pTINCR in a panel of mouse (stomach, uterus and sebaceous gland) (**b**) and human (sweat and sebaceous gland) (**c**) single epithelial tissues using a polyclonal custom-made antibody. Size bar is 100 µm.

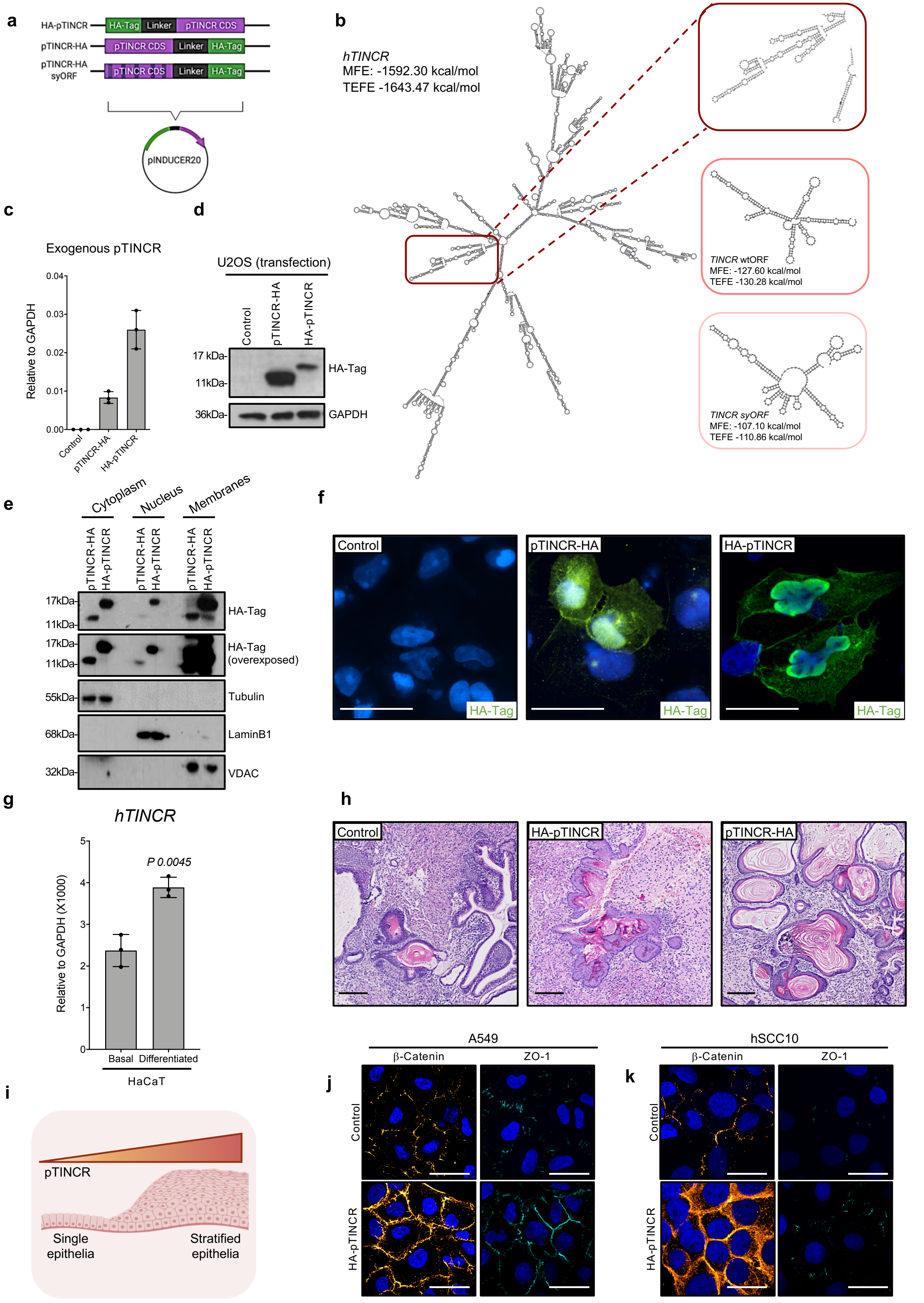
a
HA-pTINCR
HA-Tag
Linker
pTINCR CDS
pTINCR-HA
pTINCR-HA syORF
pINDUCER20
b
hTINCR
MFE: -1592.30 kcal/mol
TEFE -1643.47 kcal/mol
TINCR wtORF
MFE: -127.60 kcal/mol
TEFE -130.28 kcal/mol
TINCR syORF
MFE: -107.10 kcal/mol
TEFE -110.86 kcal/mol
c
Exogenous pTINCR
Relative to GAPDH
Control
pTINCR-HA
HA-pTINCR
d
U2OS (transfection)
17 kDa
11kDa
36kDa
HA-Tag
GAPDH
e
Cytoplasm
Nucleus
Membranes
17kDa
11kDa
HA-Tag (overexposed)
55kDa
Tubulin
68kDa
LaminB1
32kDa
VDAC
f
g
hTINCR
P 0.0045
Relative to GAPDH (X1000)
Basal
Differentiated
HaCaT
h
i
pTINCR
Single epithelia
Stratified epithelia
j
k
A549
hSCC10
β-Catenin
ZO-1

Supplementary Fig. 2 | Generation of gain-of-function tools to assess pTINCR function on epithelial differentiation.
a. Diagram representing the HA-tagged pTINCR constructs cloned in an inducible lentiviral vector.
b. Minimum Free Energy (MFE) structure of the human *TINCR* lncRNA, *TINCR* wtORF and *TINCR* syORF, calculated using RNAfold web server from ViennaRNA web.
c-f. Exogenous pTINCR expression was induced by doxycycline in U2OS cells transiently transfected with C-terminal (pTINCR-HA) and N-terminal (HA-pTINCR) HA-tagged microprotein. Exogenous pTINCR was detected by (**c**) RT-qPCR and (**d**) Western blot using an anti-HA antibody. (**e**) Detection of exogenous pTINCR by Western blot after subcellular fractionation. Enrichment in cytosol, nucleus and cell membranes was verified using Tubulin, LaminB1 and VDAC, respectively. (**f**) Representative immunofluorescence of exogenous pTINCR using an anti-HA antibody (green) in control and induced pTINCR cells. Error bars in (**c**) represent the mean ± SD of N=3 technical replicates from a representative experiment performed 3 times independently obtaining similar results.
g. Expression of *TINCR* transcript in basal or differentiated HaCaT cells measured by RT-qPCR. Error bars represent the mean ± SD in N=3 independent experiments. Two-sided T-TEST was performed.
h. Representative images of hematoxylin and eosin (H&E) stainings of teratoma areas with skin differentiation, as seen by the formation of keratin pearls. Size bar is 200 µm. The staining was performed in N=18 teratomas in control group; N=21 teratomas in pTINCR-HA group and N=12 teratomas in HA-pTINCR group.
i. Diagram illustrating the upregulation of pTINCR in the transition from single to stratified epithelia. Image was created with BioRender.com.
Size bar is 50µm.
j and k. Immunostainings of β-catenin (in orange) and ZO-1 (in blue) after 4 days of doxycycline induction of HA-pTINCR microprotein in A549 (**h**) and hSCC10 (**i**) cells. Nuclei are counterstained with DAPI. Immunostainings were performed independently 3 times obtaining the same results.

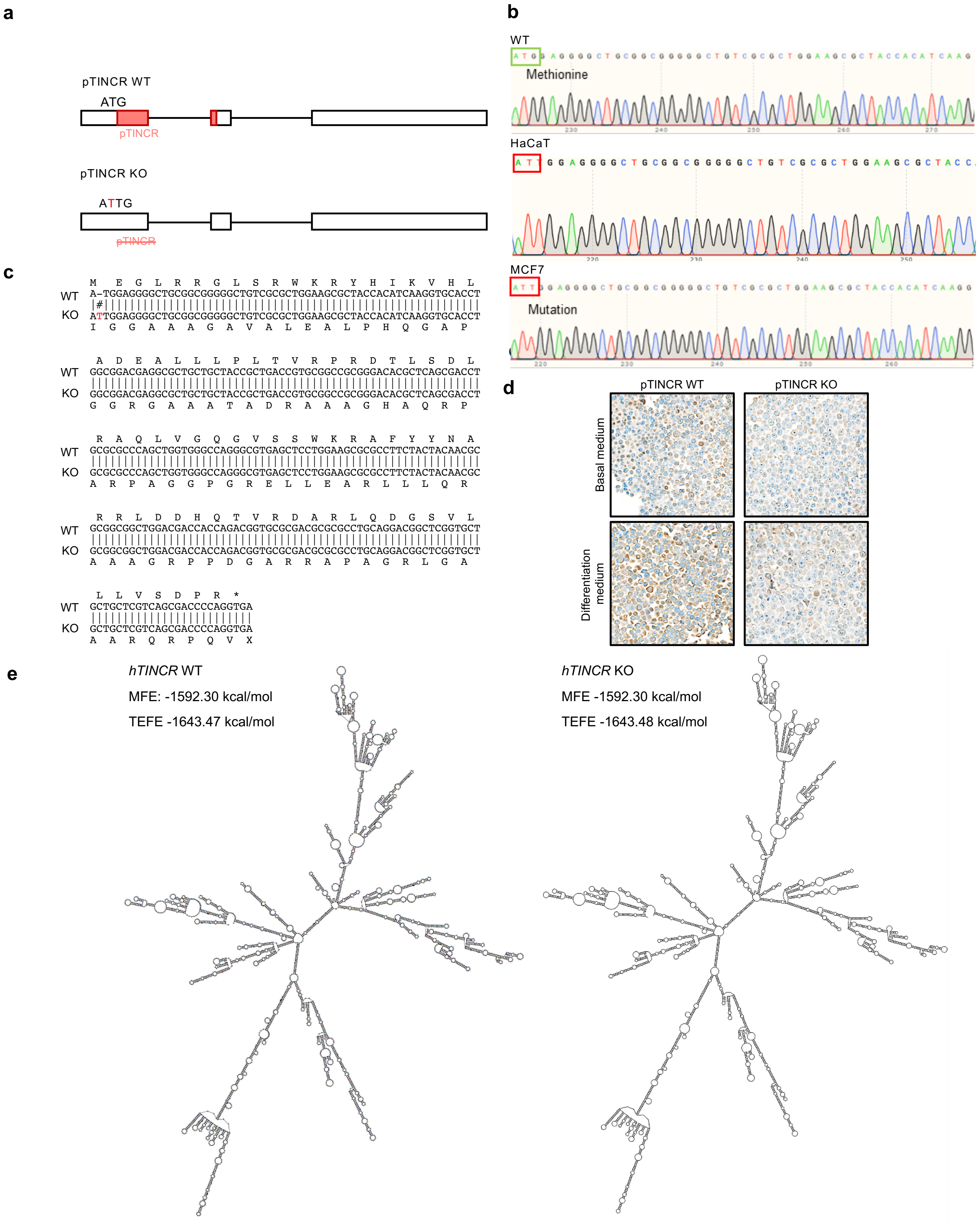

Supplementary Fig. 3 | Generation of pTINCR-KO cell lines by CRISPR-Cas9 technology.

a. Schematic representation of the WT and pTINCR-KO locus, in which a single nucleotide insertion (T) was generated by CRISPR-Cas9 technology, thereby disrupting pTINCR translation.

b. Sequencing of pTINCR locus in WT and pTINCR-KO cell lines. The editing of the start codon is highlighted.

c. Alignment of WT and pTINCR-KO nucleotide sequence. Hashtags mark differences between the two sequences.

d. Representative image of an IHC staining of WT and pTINCR-KO MCF7 cells cultured in basal and differentiation media using a pTINCR antibody.

e. Minimum Free Energy (MFE) structure of *hTINCR* WT and *hTINCR* KO lncRNA, using RNAfold web server from ViennaRNA web.

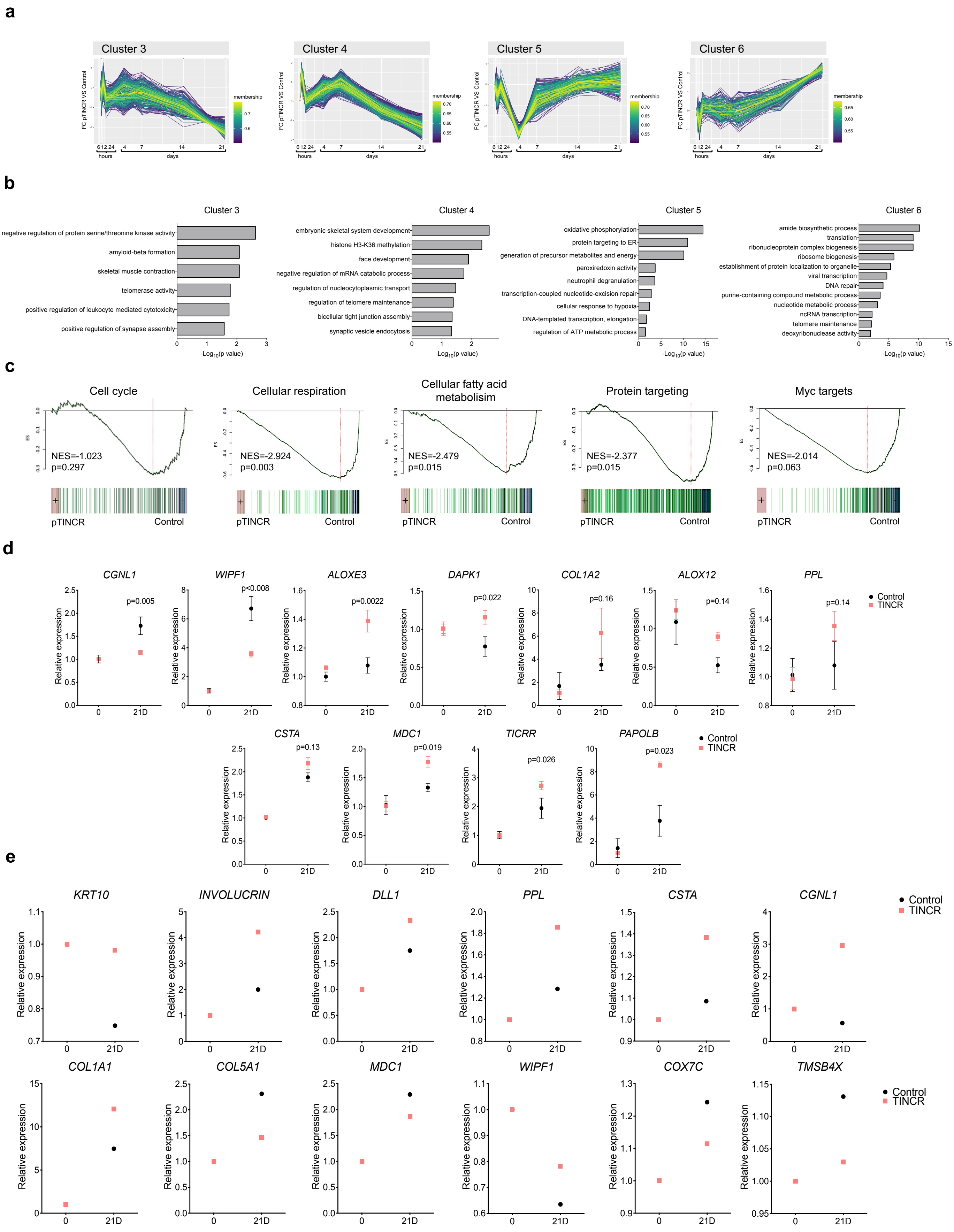
a
Cluster 3
Cluster 4
Cluster 5
Cluster 6
FC pTINCR VS Control
membership
hours
days
b
Cluster 3
negative regulation of protein serine/threonine kinase activity
amyloid-beta formation
skeletal muscle contraction
telomerase activity
positive regulation of leukocyte mediated cytotoxicity
positive regulation of synapse assembly
Cluster 4
embryonic skeletal system development
histone H3-K36 methylation
face development
negative regulation of mRNA catabolic process
regulation of nucleocytoplasmic transport
regulation of telomere maintenance
bicellular tight junction assembly
synaptic vesicle endocytosis
Cluster 5
oxidative phosphorylation
protein targeting to ER
generation of precursor metabolites and energy
peroxiredoxin activity
neutrophil degranulation
transcription-coupled nucleotide-excision repair
cellular response to hypoxia
DNA-templated transcription, elongation
regulation of ATP metabolic process
Cluster 6
amide biosynthetic process
translation
ribonucleoprotein complex biogenesis
ribosome biogenesis
establishment of protein localization to organelle
viral transcription
DNA repair
purine-containing compound metabolic process
nucleotide metabolic process
ncRNA transcription
telomere maintenance
deoxyribonuclease activity
-Log10(p value)
c
Cell cycle
NES=-1.023
p=0.297
Cellular respiration
NES=-2.924
p=0.003
Cellular fatty acid metabolisim
NES=-2.479
p=0.015
Protein targeting
NES=-2.377
p=0.015
Myc targets
NES=-2.014
p=0.063
pTINCR
Control
d
CGNL1 p=0.005
WIPF1 p<0.008
ALOXE3 p=0.0022
DAPK1 p=0.022
COL1A2 p=0.16
ALOX12 p=0.14
PPL p=0.14
CSTA p=0.13
MDC1 p=0.019
TICRR p=0.026
PAPOLB p=0.023
Relative expression
0
21D
Control
TINCR
e
KRT10
INVOLUCRIN
DLL1
PPL
CSTA
CGNL1
COL1A1
COL5A1
MDC1
WIPF1
COX7C
TMSB4X
Relative expression
Control
TINCR

Supplementary Fig. 4 | pTINCR-induced transcriptional program analyzed by RNA-seq.
a. Transcriptional dynamics analyzed using impulseDE, and classified in clusters according to their similar expression dynamics. Graphs represent cluster 3 to 6.
b. GO terms enrichment analysis using ClueGO software indicating the functional term enrichments of the identified genes in each cluster. Plots show significant GO term enrichment. $pV \leq 0.05$, using two-sided hypergeometric test with Bonferroni correction.
c. GSEA of pTINCR-induced genes versus indicated gene signatures.
d. Validation of deregulated genes by RT-qPCR. Plots show gene expression interaction between condition 0h and 21 days of pTINCR induction. mRNA expression is normalized to GAPDH in each sample and relative to the respective 0h condition. Error bars represent the mean ± SEM of N=3 independent experiments. Two-sided T-TEST was used as statistical method, absolute p-values are shown.
e. Validation of deregulated genes performed as described in (**d**) in another independent experiment.

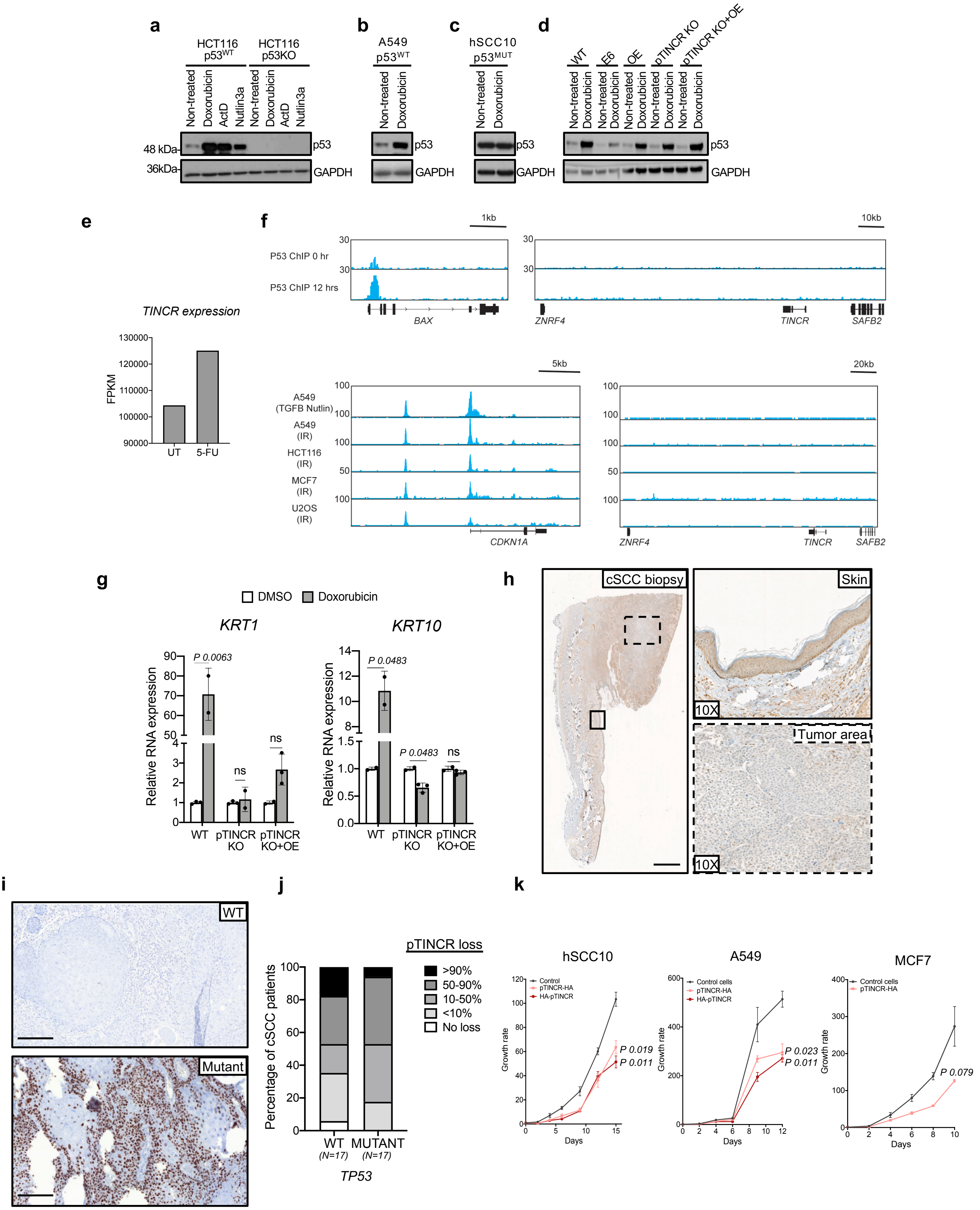

Supplementary Fig. 5 | pTINCR acts as a tumor suppressor in cSCCs.
a-d. Western blot of p53 after 24 hours of treatment with doxorubicin, actinomycin D, nutlin-3a or UV light exposure in the indicated cell lines. The experiment was performed independently at least twice in all the cell lines.
e. *TINCR* expression in HCT116 cells untreated (0hr) or treated with 5-FU (12 hrs). Data was extracted from GSE58506.
f. ChIP-seq analysis of p53 upon damage. Upper panel: HCT116 cells untreated (0 hr) or treated with 5-FU for 12 hours (12 hrs). Screenshots of p53 binding to *BAX* locus (left) and *TINCR* locus (right). Data was extracted from GSE58506. Bottom panel: A549, HCT116, MCF7 and U2OS cells treated with TGF-β and nutlin or irradiated (IR). Screenshots of p53 binding to *CDKN1A* (left) and *TINCR* (right) locus. Data was extracted from GSE100292.
g. Expression of the indicated differentiation markers by RT-qPCR after 24 hours of treatment with doxorubicin in WT, pTINCR-KO or pTINCR-KO expressing pTINCR-HA (pTINCR KO+OE) MCF7 cells. mRNA expression is normalized to GAPDH and relative to the control in each case. Error bars represent the mean ± SD in N=3 technical replicates from a representative experiment performed 3 times independently with similar results. n.s: not significant. Multiple T-TEST corrected for multiple comparison was performed.
h. pTINCR expression was analyzed by IHC in a cSCC patient cohort (N=51). Representative images showing the loss of pTINCR expression in cSCC compared with healthy epidermis to cSCC (left) and the magnification of healthy skin and tumor areas (right). Size bar is 500µm.
i. Representative IHC images of WT and TP53 mutant cSCC tumors stained for p53 (N=41). Size bar is 200µm.
j. Distribution of pTINCR loss in cSCC tumors (N=34) with wild-type (WT) or mutant TP53.
k. Growth curves of hSCC10, A549 and MCF7 cells expressing pTINCR or not. Graph represents the cell number at each time point relative to the starting number of cells (day 0). Error bars represent the mean ± SD of N=3 technical replicates from a representative experiment performed independently 3 times (A549 and hSSC10) or twice (MCF7) with similar results. Two-sided T-TEST was used as statistical method.

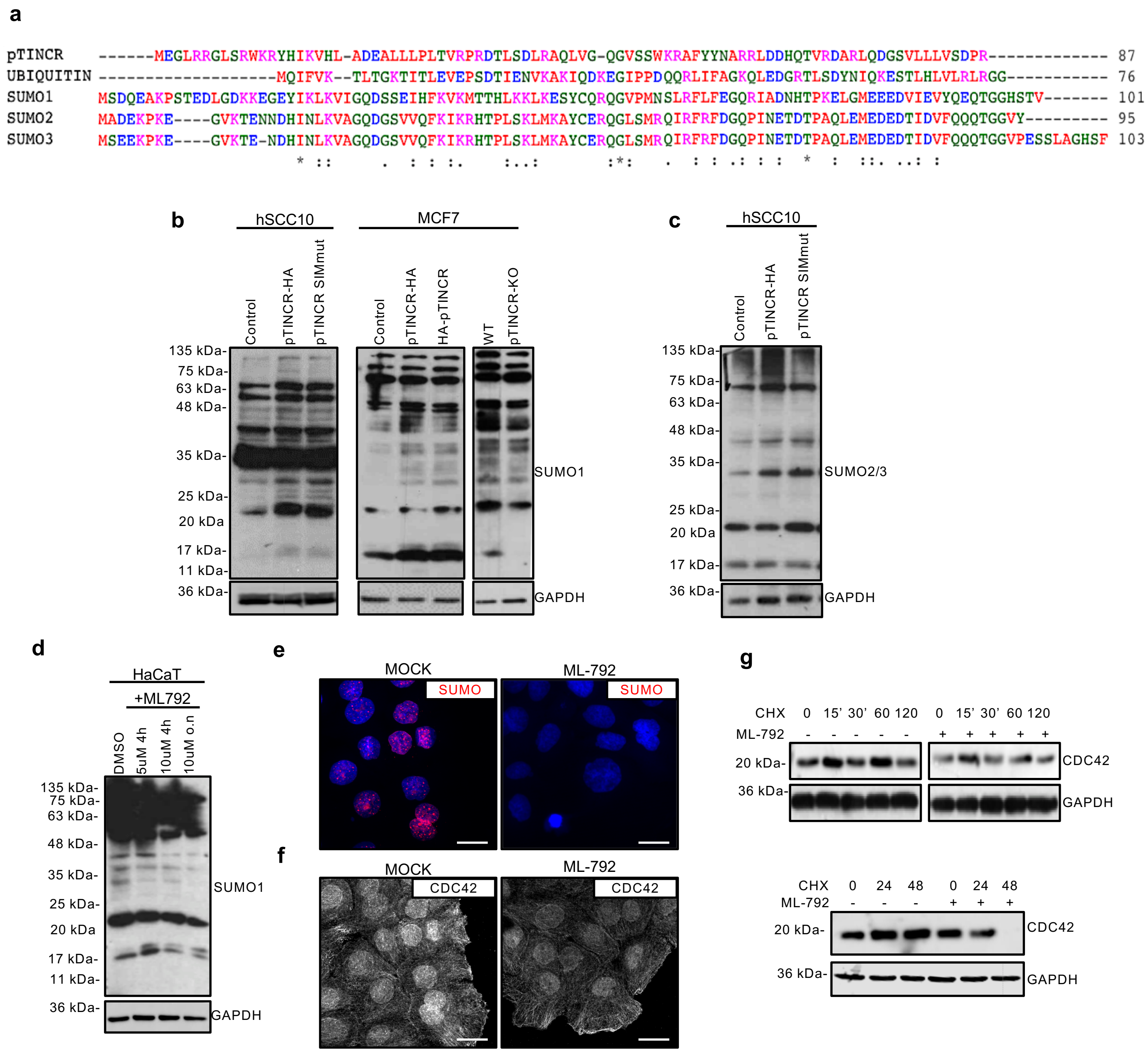

Supplementary Fig. 6 | Molecular studies to analyze pTINCR/SUMO/CDC42 interconnection.

a. Sequence alignment of human pTINCR with ubiquitin and SUMO family by multiple sequence alignment performed by Clustal Omega. The colors of the amino acid residues indicate their properties (pink, positive charge; blue, negative charge; red, hydrophobic; green, hydrophilic). The symbols below the alignment represent the biochemical similarity of aligned amino acids, with asterisks indicating identical conservation, colons representing high similarity, and periods showing somewhat similar conservation.

b and c. Effect of pTINCR overexpression and pTINCR deficiency in SUMO1 (**b**) and SUMO2/3 (**c**) conjugation analyzed by Western blot in the indicated cell lines. In MCF7, we used the syORF construct for pTINCR overexpression. Immunoblotings were performed independently 3 times (hSCC10) or 2 times (MCF7) obtaining the same results (**b**) and 1 time (**c**).

d. Western blot of SUMO1 in HaCaT cells treated or not with the SUMOylation inhibitor ML-792 for the indicated times. Immunoblotings were performed independently 3 times obtaining similar results.

e. Immunostaining images showing SUMO1 staining in HaCaT cells treated or not with ML-792 for 16 hours. Immunobloting has been performed once. Scale bar is 20µm.

f. Immunostaining images showing CDC42 staining in HaCaT cells treated or not with ML-792 for 16 hours. Immunobloting has been performed once. Scale bar is 20µm.

g. Analysis of CDC42 stability in HaCaT cells upon SUMOylation inhibition. Cells were treated with ML-792 for 16 hours prior CHX treatment for the indicated time points and protein levels were analyzed by Western blot analysis. Immunobloting has been performed twice.

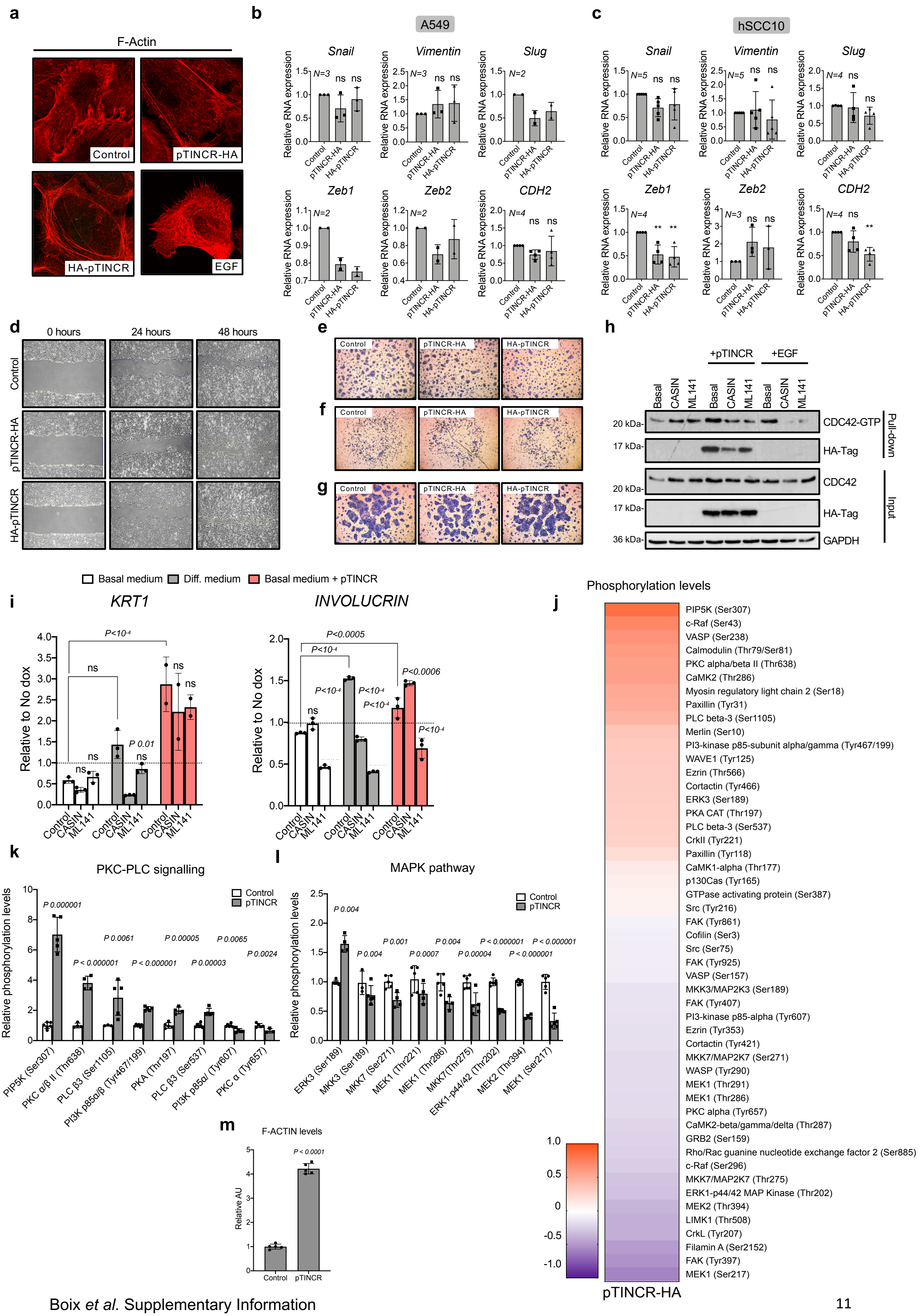

a
F-Actin
Control
pTINCR-HA
HA-pTINCR
EGF
b
A549
Snail
Vimentin
Slug
Zeb1
Zeb2
CDH2
Relative RNA expression
Control
pTINCR-HA
HA-pTINCR
c
hSCC10
d
0 hours
24 hours
48 hours
e
f
g
h
+pTINCR
+EGF
Basal
CASIN
ML141
CDC42-GTP
HA-Tag
CDC42
GAPDH
Pull-down
Input
20 kDa-
17 kDa-
36 kDa-
Basal medium
Diff. medium
Basal medium + pTINCR
i
KRT1
INVOLUCRIN
Relative to No dox
j
Phosphorylation levels
PIP5K (Ser307)
c-Raf (Ser43)
VASP (Ser238)
Calmodulin (Thr79/Ser81)
PKC alpha/beta II (Thr638)
CaMK2 (Thr286)
Myosin regulatory light chain 2 (Ser18)
Paxillin (Tyr31)
PLC beta-3 (Ser1105)
Merlin (Ser10)
PI3-kinase p85-subunit alpha/gamma (Tyr467/199)
WAVE1 (Tyr125)
Ezrin (Thr566)
Cortactin (Tyr466)
ERK3 (Ser189)
PKA CAT (Thr197)
PLC beta-3 (Ser537)
CrkII (Tyr221)
Paxillin (Tyr118)
CaMK1-alpha (Thr177)
p130Cas (Tyr165)
GTPase activating protein (Ser387)
Src (Tyr216)
FAK (Tyr861)
Cofilin (Ser3)
Src (Ser75)
FAK (Tyr925)
VASP (Ser157)
MKK3/MAP2K3 (Ser189)
FAK (Tyr407)
PI3-kinase p85-alpha (Tyr607)
Ezrin (Tyr353)
Cortactin (Tyr421)
MKK7/MAP2K7 (Ser271)
WASP (Tyr290)
MEK1 (Thr291)
MEK1 (Thr286)
PKC alpha (Tyr657)
CaMK2-beta/gamma/delta (Thr287)
GRB2 (Ser159)
Rho/Rac guanine nucleotide exchange factor 2 (Ser885)
c-Raf (Ser296)
MKK7/MAP2K7 (Thr275)
ERK1-p44/42 MAP Kinase (Thr202)
MEK2 (Thr394)
LIMK1 (Thr508)
CrkL (Tyr207)
Filamin A (Ser2152)
FAK (Tyr397)
MEK1 (Ser217)
pTINCR-HA
k
PKC-PLC signalling
Relative phosphorylation levels
Control
pTINCR
PIP5K (Ser307)
PKC α/β II (Thr638)
PLC β3 (Ser1105)
PI3K p85α/β (Tyr467/199)
PKA (Thr197)
PLC β3 (Ser537)
PI3K p85α/ (Tyr607)
PKC α (Tyr657)
l
MAPK pathway
ERK3 (Ser189)
MKK3 (Ser189)
MKK7 (Ser271)
MEK1 (Thr221)
MEK1 (Thr286)
MKK7(Thr275)
ERK1-p44/42 (Thr202)
MEK2 (Thr394)
MEK1 (Ser217)
m
F-ACTIN levels
Relative AU
P < 0.0001

Supplementary Fig. 7 | Molecular studies of pTINCR-induced CDC42 activation.

a. Effect of pTINCR or EGF in actin-cytoskeleton. hSCC10 cells were treated with EGF for 5 min or induced with doxycycline for 24 hours to induce pTINCR expression and immunofluorescence was performed using Phalloidin-TRITC antibody. Images magnification is 100X.

b and c. Expression of the indicated EMT markers in A549 (**b**) and hSCC10 (**c**) cells where expression of pTINCR have been induced with doxycycline for 4 days, measured by RT-qPCR. mRNA expression is normalized to GAPDH and relative to the control in each case. Error bars represent the mean ± SD. N is indicated in the figure for each experiment. Statistical analysis was performed using a 2-way ANOVA test corrected for multiple comparison.

d. Cell migration was assessed by wound healing assay in A549 cells. Cells were treated with doxycycline for 4 days to induce pTINCR expression before performing the scratch on the monolayer. Figure shows representative pictures of migrating cells 24 and 48 hours post-scratch.

e. Cell migration in hSCC10 was assessed by transwell assay. Cells were treated with doxycycline for 4 days to induce pTINCR expression before seeded in transwells. Figure shows representative pictures of migrating cells 24 hours post-seeding.

f and g. Cell invasion was assessed using Matrigel-covered transwells in A549 (**f**) and hSCC10 (**g**) cell lines. Cells were treated with doxycycline for 4 days to induce pTINCR expression before seeded in transwells. Figure shows representative pictures of invading cells 24 hours post-seeding.

h. CDC42 activation assay in HaCaT cells performed by pulling down GTP-CDC42 followed by Western blot using an anti-CDC42 antibody. Cells were treated with the CDC42 inhibitors CASIN or ML141 for 16 hours and then CDC42 activation was induced by doxycycline treatment for 24h (pTINCR overexpression) or EGF treatment 5 minutes before protein collection.

i. Expression of the indicated differentiation markers upon 24 hours of calcium-induced-differentiation or pTINCR-HA syORF overexpression in HaCaT cells cultured in basal medium, treated or not with CASIN or ML-141. Error bars represent the mean ± SD of N=3 technical replicates from a representative experiment performed 3 times independently with similar results. We show statistical differences within each experimental condition and between basal control cells and differentiated control cells or pTINCR-overexpressing cells (shown in brackets). n.s: not significant. 2-way ANOVA test with multiple comparison was performed.

j. Heatmap representing phosphorylation levels of proteins associated with actin cytoskeleton dynamics upon pTINCR overexpression in hSCC10 after 4 days of doxycycline induction. Values are normalized first to the loading control and second to the total amount of each protein.

k and l. Phosphorylation levels of PKC-PLC (**k**) and MAPK (**l**) pathway-related proteins after 4 days of pTINCR overexpression in hSCC10. Data are normalized first to the loading control and second to the total amount of each protein. Values are represented in the graph relativized to the control. Error bars represent the mean ± SD of N > 3 technical replicates. Statistical analysis was performed using using Multiple T-TEST corrected for multiple comparison.

m. Relative protein levels of total F-ACTIN after 4 days of pTINCR overexpression in hSCC10. Data are normalized to the loading control. Represented values are relativized to the control. Error bars represent the mean ± SD of 5 technical replicates. Two-sided T-TEST was performed.

Supplementary Table 1: PhyloCSF analysis of lncRNAs enriched in skin

	Skin enrichment score	PhyloCSF score
LINC00302	0.9999	0
RP13-455A7.1	0.9931	0
CHODL-AS1	0.9807	0
RP11-73G16.21	0.9357	0
RP5-1121H13.4	0.9331	0
LINC01698	0.9075	0
RP4-529N6.2	0.8569	0
RP11-252C15.1	0.819	0
AC007389.3	0.762	0
RP11-55K22.5	0.7388	0
KRT73-AS1	0.6949	188.678
LINC00393	0.6183	0
RP11-191L9.4	0.5912	0
KB-1410C5.3	0.5731	0
SCGB1B2P	0.5704	0
RP5-884C9.2	0.5348	0
RP4-737E23.2	0.5111	0
RP11-521D12.5	0.5083	0
TINCR	0.508	675.745
GRM7-AS1	0.4768	0
RP11-60A8.1	0.4715	0
RP11-111F5.4	0.4402	0
LINC00640	0.4385	0
CTD-3247F14.2	0.4371	0
LRP4-AS1	0.4019	377.158
KB-1930G5.4	0.3684	0
RP11-424M21.1	0.3552	0
CTD-3032H12.2	0.3542	0
LINC02660	0.3518	0
RP11-111F5.3	0.3294	0
LINC00504	0.3213	0
WI2-85898F10.1	0.3086	0
SOX21-AS1	0.2845	0
LINC02518	0.2843	0
LINC02541	0.2813	0
RP11-304F15.4	0.2779	0
AC019064.1	0.277	0
RP11-804N13.1	0.2766	0
RP11-365O16.3	0.2682	0
LINC00842	0.2606	0
CALML3-AS1	0.2523	0
RP11-646E18.4	0.249	0
HOTAIR	0.2433	177.776
RP11-304F15.6	0.2355	0
LINC01254	0.2326	0
CTB-49A3.2	0.2235	0
RP11-115D19.1	0.2197	0
RP11-146I2.1	0.2165	0
RP11-84E24.2	0.2148	0
RP11-366M4.3	0.2147	85.609

Supplementary Table 2: Constructs for pTINCR exogenous expression.

In capital letters, pTINCR sORF; in italics, linker sequence; in bold, HA-Tag sequence.

Name	Backbone	ORF
**HA-pTINCR**	pInducer20	ATG**tatccttatgatgtgcctgattatgct***ggcggtggaggcagcggaggtgggggaagtggtggcggtggaagc*GAGGGGCTGC<br>GGCGGGGGCTGTCGCGCTGGAAGCGCTACCACATCAAGGTGCACCTGGCGGACGAGGCGCTGC<br>TGCTACCGCTGACCGTGCGGCCGCGGGACACGCTCAGCGACCTGCGCGCCCAGCTGGTGGGCC<br>AGGGCGTGAGCTCCTGGAAGCGCGCCTTCTACTACAACGCGCGGCGGCTGGACGACCACCAGAC<br>GGTGCGCGACGCGCGCCTGCAGGACGGCTCGGTGCTGCTGCTCGTCAGCGACCCCAGGTGA
**pTINCR-HA**	pInducer20	ATGGAGGGGCTGCGGCGGGGGCTGTCGCGCTGGAAGCGCTACCACATCAAGGTGCACCTGGCG<br>GACGAGGCGCTGCTGCTACCGCTGACCGTGCGGCCGCGGGACACGCTCAGCGACCTGCGCGCC<br>CAGCTGGTGGGCCAGGGCGTGAGCTCCTGGAAGCGCGCCTTCTACTACAACGCGCGGCGGCTGG<br>ACGACCACCAGACGGTGCGCGACGCGCGCCTGCAGGACGGCTCGGTGCTGCTGCTCGTCAGCG<br>ACCCCAGG*ggcggtggaggcagcggaggtgggggaagtggtggcggtggaagctat***ccttatgatgtgcctgattatgct**TGA
**pTINCR-HA (syORF)**	pInducer20	ATGGAGGGCCTGAGAAGAGGCCTGAGCAGATGGAAGAGATACCACATCAAGGTGCACCTGGCCG<br>ACGAGGCCCTGCTGCTGCCACTGACAGTGAGGCCCAGGGACACCCTGAGCGACCTGAGAGCCCA<br>GCTGGTGGGCCAGGGCGTGAGCTCTTGGAAGAGAGCCTTCTACTACAACGCCAGGAGGCTGGAC<br>GACCACCAGACAGTGAGGGATGCCAGGCTGCAGGACGGCAGCGTGCTGCTGCTGGTGTCCGACC<br>CCAGG*ggcggaggaggaagcggaggaggaggctccggcggaggaggttcctac***ccttacgacgtgcctgactacgcc**TGA
**pTINCR-SIMmut (syORF)**	pInducer20	ATGGAGGGCCTGAGAAGAGGCCTGAGCAGATGGAAGAGATACCACATCAAGGTGCACCTGGCCG<br>ACGAGGCCCTGCTGCTGCCACTGACAGTGAGGCCCAGGGACACCCTGAGCGACCTGAGAGCCCA<br>GCTGGTGGGCCAGGGCGTGAGCTCTTGGAAGAGAGCCTTCTACTACAACGCCAGGAGGCTGGAC<br>GACCACCAGACAGTGAGGGATGCCAGGCTGCAGGACGGCAGCGCCGCTGCTGCCGTGTCCGAC<br>CCCAGG*ggcggaggaggaagcggaggaggaggctccggcggaggaggttcctac***ccttacgacgtgcctgactacgcc**TGA

Supplementary Table 3: CRAPome analysis of pTINCR interactors

	FC-B	SAINT probability
CDC42	6.45	1
HNRNPC	6.42	1
BANF1	5.14	1
CPSF6	2.53	1
EEF2	2.48	1
EIF4B	2.47	1
GRSF1	2.19	1
DNAJA2	2.19	1
RPL11	2.06	1
KRT18	1.99	1
HIST1H2BN	0.01	0.68
CCT3	0.54	1
GPI	0.36	1
RPS3	0.09	1
RPLP0	0.02	0.51
MYL12B	0.27	0.35
RPS2	0.02	0.49
RPL7A	0.07	0.86
RPL10A	0.06	1
RPL3	0.04	1
HNRNPA1	0.02	1
RPL18	0.01	1
ETFA	0.58	0
RPL17-C18ORF32	0.01	0
ILF3	0.01	0.76
RPL7	0.01	1
FXR2	0.54	0
RPL23	0.01	0
NONO	0.06	0.0037
RPL23A	0.02	0
MATR3	0.06	0
RPS24	0.22	0.46
AGR2	0.17	0
EIF1	0.17	0
YBX1	0	0

Supplementary Table 4: Phosphorylation levels of cytoskeleton-related proteins

	Control		pTINCR	
Proteins	Ph_{levels}	SD (n=6)	Ph_{levels}	SD (n=6)
PLC beta-3 (Ser1105)	2.67	0.10	7.58	3.10
VASP (Ser238)	1.28	0.14	5.65	1.33
c-Raf (Ser43)	0.95	0.05	4.97	1.64
PIP5K (Ser307)	0.48	0.10	3.36	0.55
PKC alpha/beta II (Thr638)	1.01	0.16	3.87	0.46
Myosin regulatory light chain 2 (Ser18)	1.02	0.08	3.37	0.33
Merlin (Ser10)	1.69	0.09	3.78	0.39
WAVE1 (Tyr125)	1.89	0.41	3.93	0.92
PLC beta-3 (Ser537)	1.99	0.28	3.81	0.44
CaMK2 (Thr286)	0.61	0.13	2.27	0.56
Ezrin (Thr566)	1.50	0.07	3.08	0.11
Paxillin (Tyr31)	0.71	0.14	2.25	0.27
PI3-kinase p85-subunit alpha/gamma (Tyr467/199)	1.31	0.10	2.82	0.18
Cortactin (Tyr466)	1.34	0.15	2.72	0.31
Calmodulin (Thr79/Ser81)	0.40	0.05	1.61	0.22
ERK3 (Ser189)	0.61	0.02	1.23	0.35
p130Cas (Tyr165)	2.04	0.02	2.60	0.44
CrkII (Tyr221)	0.52	0.06	1.00	0.18
Paxillin (Tyr118)	0.49	0.01	0.80	0.15
CaMK1-alpha (Thr177)	0.88	0.06	1.19	0.27
Src (Tyr216)	0.83	0.05	0.99	0.09
PKA CAT (Thr197)	0.13	0.02	0.26	0.03
GTPase activating protein (Ser387)	0.59	0.03	0.72	0.09
FAK (Tyr861)	0.30	0.01	0.26	0.01
Src (Ser75)	0.68	0.02	0.55	0.02
MEK1 (Thr286)	0.43	0.07	0.28	0.04
Cofilin (Ser3)	1.06	0.05	0.87	0.09
FAK (Tyr925)	1.17	0.11	0.94	0.18
PI3-kinase p85-alpha (Tyr607)	0.85	0.09	0.61	0.15
Cortactin (Tyr421)	0.91	0.19	0.63	0.05
PKC alpha (Tyr657)	0.89	0.12	0.59	0.11
LIMK1 (Thr508)	0.53	0.06	0.22	0.05
c-Raf (Ser296)	0.79	0.05	0.45	0.07
FAK (Tyr407)	1.43	0.16	1.03	0.19
CrkL (Tyr207)	0.69	0.15	0.28	0.03
MKK3/MAP2K3 (Ser189)	1.51	0.25	1.09	0.12
Rho/Rac guanine nucleotide exchange factor 2 (Ser885)	1.05	0.16	0.62	0.22
WASP (Tyr290)	1.38	0.08	0.93	0.18
CaMK2-beta/gamma/delta (Thr287)	1.20	0.09	0.74	0.14
ERK1-p44/42 MAP Kinase (Thr202)	1.00	0.07	0.50	0.03
VASP (Ser157)	2.68	0.16	2.15	0.24
MEK2 (Thr394)	0.94	0.04	0.41	0.08
Filamin A (Ser2152)	0.99	0.08	0.34	0.17
FAK (Tyr397)	1.16	0.36	0.35	0.06
MEK1 (Thr291)	2.53	0.33	1.68	0.13
MKK7/MAP2K7 (Ser271)	2.64	0.27	1.79	0.27
GRB2 (Ser159)	2.55	0.17	1.55	0.48
MKK7/MAP2K7 (Thr275)	2.33	0.25	1.21	0.21
MEK1 (Ser217)	1.57	0.20	0.43	0.06
Ezrin (Tyr353)	8.26	0.46	5.81	0.44

Supplementary Table 5: Mouse primers

Primer Name	Forward	Reverse
mGAPDH	TGTGTCCGTCGTGGATCTGA	TTGCTGTTGAAGTCGCAGGAG
mTINCR	TACGAACAGAACAGGAGGAC	CTCTCCACATTGTGGCTTTG

Supplementary Table 6: Human primers

Primer Name	Forward	Reverse
GAPDH	GGACTCATGACCACAGTCCATGCC	TCAGGGATGACCTTGCCCACAG
lncRNA TINCR	GGTGCTGCTGCTCGTCAG	TTCCTTCAGCCAGTACCCAG
EXO HA-pTINCR	CTTATGATGTGCCTGATTATGC	CACGGTCAGCGGTAGCAG
EXO pTINCR-HA	GGTGCTGCTGCTCGTCAG	TCAGGCACATCATAAGGATAGC
KERATIN 14	GACCATTGAGGACCTGAGGA	AGACGGGCATTGTCAATCTG
KERATIN 5	GCTGCTGCGTGAGTACCAG	CTGGTCCAACTCCTTCTCCA
KERATIN 1	GGCAGTTCCAGCGTGAAGTTTGTT	TTCTCCGGTAAGGCTGGGACAAAT
KERATIN 10	GAGCAAGGAACTGACTACAG	CTCGGTTTCAGCTCGAATCT
INVOLUCRIN	TGCCTGAGCAAGAATGTGAG	TGCTCTGGGTTTTCTGCTTT
FILAGGRIN	CATGGCAGCTATGGTAGTGCAGA	ACCAAACGCACTTGCTTTACAGA
EPCAM	ATAACCTGCTCTGAGCGAGTG	TGCAGTCCGCAAACTTTTACTA
TP63	GACGTGTCCTTCCAGCAGTC	GGGGTCATCACCTTGATCTG
ESR1	CCCACTCAACAGCGTGTCTC	CGTCGATTATCTGAATTTGGCCT
HES1	TCAACACGACACCGGATAAAC	GCCGCGAGCTATCTTTCTTCA
KERATIN 18	TCGCAAATACTGTGGACAATGC	GCAGTCGTGTGATATTGGTGT
ID3	TCATCTCCAACGACAAAAGG	ACCAGGTTTAGTCTCCAGGAA
XBP1	TGGCCGGGTCTGCTGAGTCCG	ATCCATGGGGAGATGTTCTGG
EGFR	GGCACTTTTGAAGATCATTTTCTC	CTGTGTTGAGGGCAATGAG
ERBB2	TGTGACTGCCTGTCCCTACAA	CCAGACCATAGCACACTCGG
ITGB3	CGCTACAAGGGGGAGATGT	TACGCGTGGTACAGTTGCAG
VIMENTIN	GACAATGCGTCTCTGGCACGTCTT	TCCTCCGCCTCCTGCAGGTTCTT
CDH2	CTGCACAGATGTGGACAGGA	CCACAAACATCAGCACAAGG
SNAIL1	ACCACTATGCCGCGCTCTT	GGTCGTAGGGCTGCTGGAA
SLUG	TCGGACCCACACATTACCTT	ATGAGCCCTCAGATTTGACCT
ZEB1	GCACCTGAAGAGGACCAGAG	TGCATCTGGTGTTCCATTTT
ZEB2	CGCTTGACATCACTGAAGGA	CTTGCCACACTCTGTGCATT
CGNL1	CAGCCTAGCCCAATAAGAAACC	CAGACCCGTCTTTGCGATCT
WIPF1	AGCCTCAGAGGAACCGAATG	CGGACTTGATTGAATGGGTCTTG
ALOXE3	CCCTGGATCGGTACAGAAGTA	CTTGCGGAAGAAAGCGTAGC
DAPK1	ACGTGGATGATTACTACGACACC	TGCTTTTCTCACGGCATTTCT
CSTA	AAACCCGCCACTCCAGAAATC	CACCTGCTCGTACCTTAATGTAG
MDC1	GGGCGGCTACATATCTTTAGTG	GGCATTCGGCCTACCACATT
COL1A2	GGCCCTCAAGGTTTCCAAGG	CACCCTGTGGTCCAACAACTC
ALOX12	ATGGCCCTCAAACGTGTTTAC	GCACTGGCGAACCTTCTCA
PPL	GCTGAAGACCGAGAATCCCG	CCGCAGTAGCTCGTTGGTG
TICRR	TTCCACGAATGGAAGGAATG	CCAAGGGCTCTAGGGTGACT
PAPOLB	CCTCGCCTATCAGTCTAGCG	GGCCTGAGGGTTTCTATTAGCC
TMSB4X	GACCAGACTTCGCTCGTACTCGT	GATCTCAGCCATATCGGGTTTGT
COX7C	GGTCCGTAGGAGCCACTATGA	GTGTCTTACTACAAGGAAGGGTG
DLL1	GATTCTCCTGATGACCTCGCA	TCCGTAGTAGTGTTCGTCACA
COL5A1	GCCCGGATGTCGCTTACAG	AAATGCAGACGCAGGGTACAG

Supplementary Table 7: Antibodies

Target	Reference	Dilution WB	Dilution IF	Dilution IHC
HA-Tag	Ab9110, Abcam	1:5000		
HA-Tag	H6908, Sigma		1:150	
GAPDH	AM4300, Thermo Fisher Scientific	1:10000		
pTINCR	Custom-made antibody, Abyntek	1:50	1:200	1:500
Phalloidin-TRITC	P1951, Sigma-Aldrich		20mg/ml	
CDC42 (B8)	SC-8401, Santa Cruz	1:100	1:100	
Lamin B1	PA5-19468, Thermo Fisher Scientific	1:1000		
Tubulin	SC-32293, Santa Cruz	1:1000		
Histone 3	Ab18521, Abcam	1:10000		
VDAC	SC-390996, Santa Cruz	1:500		
ZO1	GTX108592, GeneTex		1:100	
b-Catenin	GTX633010, GeneTex		1:100	
E-Cadherin	610182, BD		1:100	
SUMO1	4930S, Cell Signaling	1:1000	1:100	
SUMO2/3	4971S, Cell Signaling	1:1000		
HIS-Tag (D3I1O) XP®	12698, Cell Signaling	1:1.000		
FLAG-Tag	F1804, Sigma	1:1000		
INVOLUCRIN (clone SY5)	MS-126 P1 (), Thermo Fisher Scientific	1:500		
p53 (DO-1)	SC-126, Santa Cruz	1:1000		
B23	SC-5564	1:1000		

Supplementary Table 8: Amplicon-seq analysis quality data

cSCC Biopsy	% of region >300 reads	% of region >140 reads	% of region <10 reads	Average Coverage	Total Coverage	Coverage AfterLength Filter	Coverage After Quality Filter	Coverage RawData
10BA117731	41,4	45,4	36,5	3940	1E+06	1317838	1356677	1696016
10BA117731	28,7	38,8	35,3	2607	7E+05	871257	908752	1214320
10BA126180	41,1	48,7	33,5	2380	6E+05	934497	983952	1212868
10BA126180	31,7	38,3	40,9	1048	3E+05	405506	421761	529684
P09A110231	16,5	28,7	34,5	366	80781	819483	1009623	1294216
P09A110231	12,7	22,8	38,1	234	53138	540815	654607	831570
P09A112459	34,8	44,9	29,2	666	2E+05	765817	889372	1104768
P09A112459	37,1	49,5	26,6	712	2E+05	544927	640924	790980
P09A119469	53,3	59,9	24,4	1574	4E+05	870414	969099	1173964
P09A119469	56,1	64,0	24,9	1733	4E+05	915918	1024772	1208380
P09A49386	25,6	28,4	47,0	933	2E+05	418790	435071	575758
P09A49386	40,6	48,5	33,0	902	2E+05	377072	405392	477078
10BA1103806	55,8	64,2	19,0	1421	4E+05	527295	609341	875314
10BA1103806	54,8	65,5	17,5	1205	3E+05	418742	487949	664142
10BA111995	54,3	57,9	26,6	3197	8E+05	1187113	1436560	1915606
10BA111995	71,3	73,9	23,1	3643	9E+05	1217833	1424410	1868750
10BA116772	58,6	60,7	28,7	3248	8E+05	1066231	1233623	1640366
10BA19615	67,5	72,3	23,1	3048	8E+05	994388	1134004	1474434
10BA19615	65,0	69,3	23,9	2162	5E+05	748034	851887	1184360
20BA3264	69,3	76,4	13,5	1458	3E+05	478022	544711	717994
20BA3264	69,0	77,7	11,9	1473	4E+05	492569	572061	738432
20BA78203	59,6	68,3	14,7	1067	3E+05	413147	543105	734982
20BA78203	56,1	67,5	13,7	1022	2E+05	413354	520882	756978
10BA116777	59,6	65,5	15,5	3684	9E+05	1151326	1347665	1683522
10BA116777	57,1	64,2	18,3	2310	6E+05	744807	899005	1123748
10BA121019	50,8	60,9	22,6	1405	4E+05	453124	529074	666890
10BA121019	63,5	68,3	19,0	3163	8E+05	1015505	1203054	1500052
20BA18207	76,9	80,7	9,9	3027	7E+05	945338	1066983	1332342
20BA18207	76,9	81,0	10,9	2597	6E+05	813805	931558	1138942
K09A118620	33,8	39,3	30,2	1518	4E+05	478876	574063	740362
K09A118620	45,2	51,0	19,8	2430	6E+05	760782	899550	1111438
P10A11765	36,5	42,1	26,6	1638	4E+05	529057	652461	832024
P10A11765	45,7	52,3	24,6	2406	6E+05	805802	999296	1287894
X09A210280	59,1	67,0	17,5	1421	4E+05	454239	531399	655062
X09A210280	66,2	71,1	17,8	3585	9E+05	1172127	1388894	1768210
20BA114488	54,8	64,7	20,1	3252	7E+05	873827	889511	1171718
20BA114488	59,1	65,2	17,8	2537	5E+05	671095	686024	913484
10BA13428	50,0	56,6	22,3	2201	5E+05	729198	747102	1016636
10BA116319	70,3	73,6	23,1	7176	2E+06	2080151	2223457	2523336
10BA116319	71,1	73,1	22,6	6818	2E+06	1911204	2034700	2278188
10BA116725	68,5	72,3	21,1	3182	8E+05	1165554	1311536	1488814
10BA116725	64,2	70,3	22,3	1973	5E+05	664178	741990	836194
10BA19901	67,5	72,1	21,8	3043	7E+05	1008148	1102391	1236968
10BA19901	58,1	68,0	23,6	1222	3E+05	392302	429528	484956
20BB11500	75,6	77,2	18,8	4011	9E+05	1169882	1257636	1426734
20BB11500	67,0	73,1	19,8	1322	3E+05	373517	399538	452030
P09A223347	61,4	69,0	23,1	4192	1E+06	1524657	1697621	1926556
P09A223347	67,8	70,6	24,6	5421	1E+06	1897908	2108179	2373294
10BA112963	84,3	86,3	9,9	4113	1E+06	1165471	1309616	1555388
10BA112963	79,2	83,8	10,4	2690	6E+05	779150	852933	1031178
P09A115715	73,9	78,9	11,2	3237	8E+05	977686	1108616	1363564
P09A115715	73,6	80,2	12,2	3311	8E+05	972420	1062215	1291264
P09A123251	81,7	85,8	10,4	4536	1E+06	1336342	1493414	1808258
P09A123251	81,2	85,5	10,4	4698	1E+06	1378736	1491018	1796348
P09A123260	73,4	76,9	12,7	5108	1E+06	1518874	1704261	2059078
P09A123260	70,1	73,1	17,8	3549	9E+05	1063426	1139312	1395742
P09A15782	73,9	80,2	12,4	2771	7E+05	812671	897048	1088228
P09A15782	77,9	82,7	10,7	4155	1E+06	1267163	1402574	1722834
P09A221036	81,2	85,0	9,9	3573	8E+05	1034362	1151474	1399720
P09A221036	81,5	85,3	9,9	4304	1E+06	1248412	1362126	1631020
P10A11079	78,9	83,5	10,4	3863	9E+05	1140876	1280963	1521942
P10A11079	73,9	81,2	12,4	3289	8E+05	975145	1078564	1289118
20BA13108	78,2	83,0	7,4	3725	9E+05	1239578	1457304	1846622
20BA13108	77,7	81,5	9,1	3944	1E+06	1352563	1571685	2026498
20BA76404	78,7	82,0	6,6	3537	9E+05	1186652	1366478	1717040
20BA76404	79,7	81,5	8,9	4668	1E+06	1532075	1772514	2207844

Supplementary Source Data of Supplementary Fig. 2

d

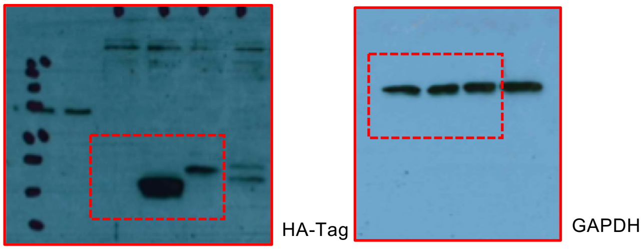

e

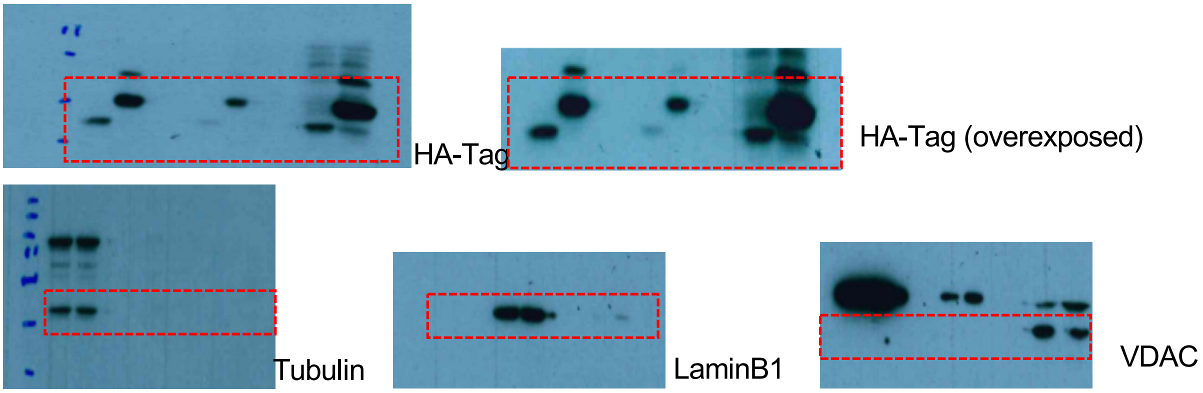

Supplementary Source Data of Supplementary Fig. 5

a

HCT116
p53^WT

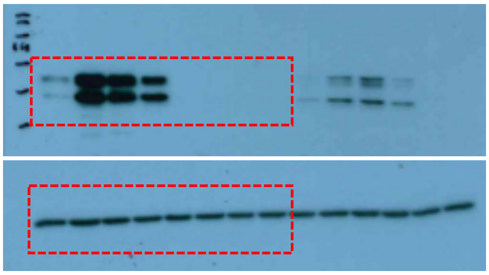

b

A549
p53^WT

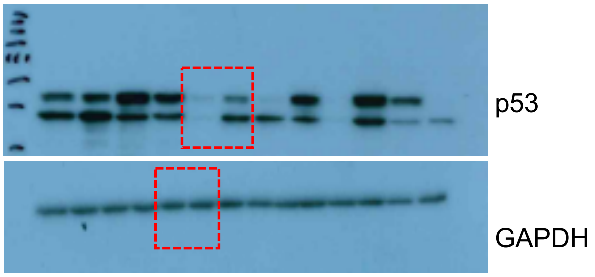

c

hSCC10

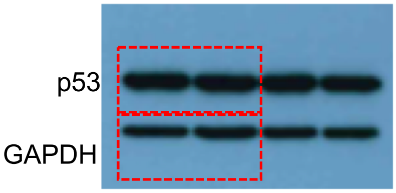

d

MCF7
p53^WT

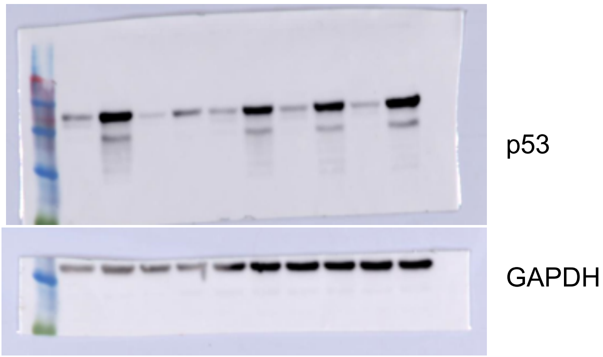

b

hSCC10 MCF7 MCF7 pTINCR KO

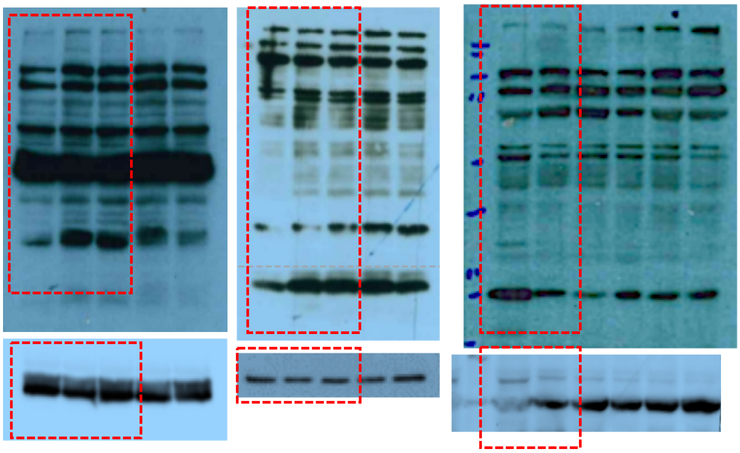

SUMO1

GAPDH

c

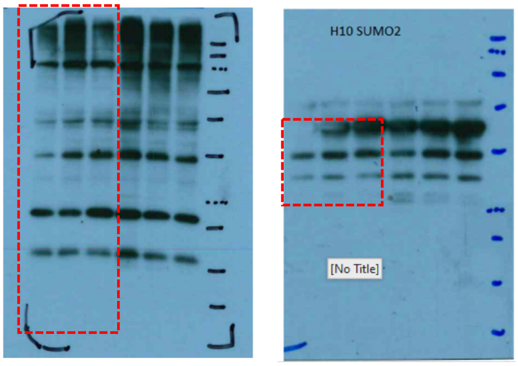

SUMO2/3 GAPDH

d

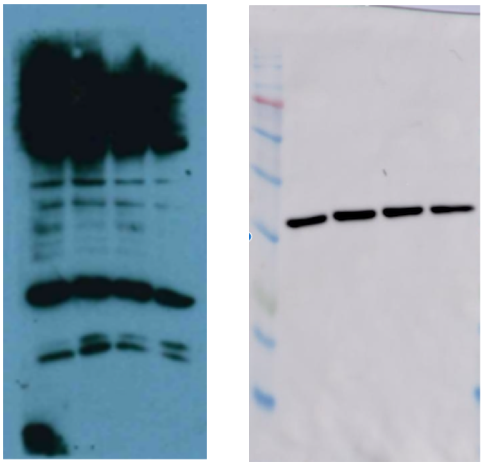

SUMO1 GAPDH

g

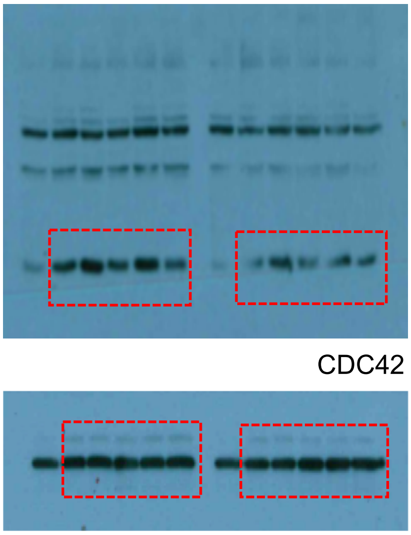

CDC42

GAPDH

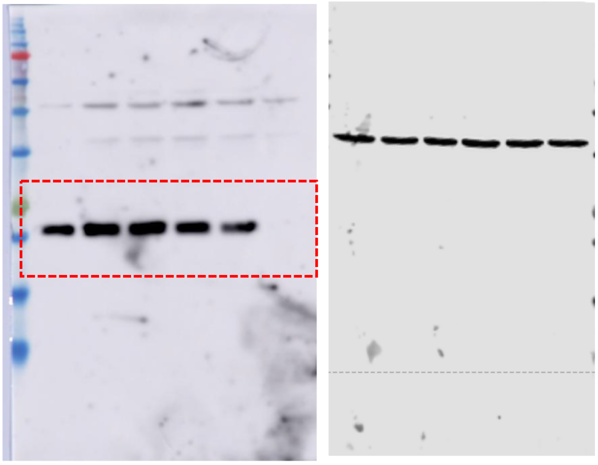

CDC42 GAPDH

Supplementary Source Data of Supplementary Fig. 7

h

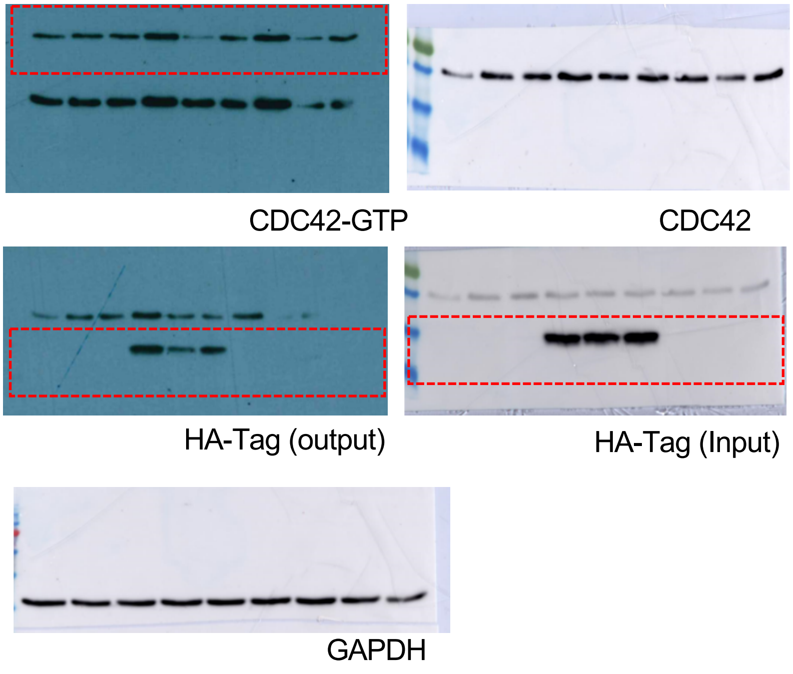

Supplement: Supplementary file 1 — Supplementary Information [file 41467_2022_34529_MOESM1_ESM.pdf]
