# Supplementary material for: pTINCR microprotein promotes epithelial differentiation and suppresses tumor growth through CDC42 SUMOylation and activation
Source: Nat Commun. 2022 Nov 11;13:6840. doi: 10.1038/s41467-022-34529-6 (PMC9652315; doi:10.1038/s41467-022-34529-6)
Supplement: Supplementary file 3 — Reporting Summary [file 41467_2022_34529_MOESM3_ESM.pdf]

nature portfolio

Corresponding author(s): María Abad
Last updated by author(s): Oct 6, 2022

Reporting Summary

Nature Portfolio wishes to improve the reproducibility of the work that we publish. This form provides structure for consistency and transparency in reporting. For further information on Nature Portfolio policies, see our Editorial Policies and the Editorial Policy Checklist.

Statistics

For all statistical analyses, confirm that the following items are present in the figure legend, table legend, main text, or Methods section.

n/a	Confirmed	
☐	☒	The exact sample size (n) for each experimental group/condition, given as a discrete number and unit of measurement
☐	☒	A statement on whether measurements were taken from distinct samples or whether the same sample was measured repeatedly
☐	☒	The statistical test(s) used AND whether they are one- or two-sided<br>*Only common tests should be described solely by name; describe more complex techniques in the Methods section.*
☒	☐	A description of all covariates tested
☐	☒	A description of any assumptions or corrections, such as tests of normality and adjustment for multiple comparisons
☐	☒	A full description of the statistical parameters including central tendency (e.g. means) or other basic estimates (e.g. regression coefficient) AND variation (e.g. standard deviation) or associated estimates of uncertainty (e.g. confidence intervals)
☐	☒	For null hypothesis testing, the test statistic (e.g. F, t, r) with confidence intervals, effect sizes, degrees of freedom and P value noted<br>*Give P values as exact values whenever suitable.*
☒	☐	For Bayesian analysis, information on the choice of priors and Markov chain Monte Carlo settings
☒	☐	For hierarchical and complex designs, identification of the appropriate level for tests and full reporting of outcomes
☒	☐	Estimates of effect sizes (e.g. Cohen's d, Pearson's r), indicating how they were calculated

Our web collection on statistics for biologists contains articles on many of the points above.

Software and code

Policy information about availability of computer code

Data collection	- qRT-PCR data were obtained using 7900HT Fast Real-Time PCR System (Applied Biosystems) and data were analyzed using GraphPad Prism (v8.4.0).<br>- Immunofluorescence images were obtained using a Nikon Eclipse Ti-E inverted microscope.<br>- RNAseq data were obtained in an The NextSeq 500™ (Illumina Inc.), with a read length of 2x76bp; alignments were performed using STAR software (v2.5.2b) with default parameters.<br>- Mass spectrometry data were collected using a an LTQ Orbitrap Velos instrument (ThermoFisher), and Progenesis ® QI software v3.0 (Nonlinear dynamics, UK) was used for MS data analysis using default settings.<br>- Phosphorylation levels of human cytoskeleton proteins were obtained by using a phosphorylation-specific antibody microarray (Fullmoon Biosystems Inc.).
Data analysis	- qRT-PCR data were analyzed using GraphPad Prism (v8.4.0).<br>- Immunofluorescence images were analyzed with Fiji (v2.0.0).<br>- Statistical analyses (excluding the sequencing data) were performed with Graph Pad Prism (v8.4.0).<br><br>For RNA-Seq Analysis of TCGA:<br>- Paired-end reads from RNA-Seq were aligned using Tophat (v2.1.0) to the human genome (hg19).<br>- Differentially expressed genes were identified using HTSeq + DESeq2 (v1.24.0).<br><br>For Ribosome profiling analysis:<br>- Reads were mapped to the assembled mouse genome (mm10) using Bowtie2 (v2.3.4.3).<br><br>For RNAseq data analysis:<br>- Data alignments were performed using STAR software (v2.5.2b) with default parameters.<br>- Sambamba (v0.6.7) was used to convert to bam and sort resulting sam files.

- Genes were annotated using biomaRt (vGRCh38.p12).
- All subsequent analyses were performed in the R programming environment (https://www.R-project.org) as detailed step-by-step in the Methods section. R packages used are: rsubread, DESeq2, ImpulseDE2, e107, ggplot2, viridis, ggExtra, gridExtra and limma. Gene ontology analysis was performed using Cytoscape (v3.9.1) and ClueGo (v2.5.7) tools.
- Gene ontology terms were computed using the ClueGO (v2.5.7) module of Cytoscape (v3.9.1) with default parameters.

For Amplicon-seq analysis:
- Initial alignment was performed with BWA (v0.7.17)
- Primer sequence clipping and variant calling was done with the GATK Unified Genotyper (v3.4.0) and VarScan2 (v2.4.3).
- Annotation was done with ANNOVAR (annovar_180416)

For LC-MS/MS identification of pTINCR microprotein:
- Progenesis ® QI for proteomics software v3.0 was used for MS data analysis using default settings.
- Files were processed with Proteome Discoverer (v2.1).

For interactome analysis and validation:
- MS data analysis was performed using Progenesis ® QI for proteomics (v3.0).
- Runs were automatically aligned with manual supervision Peak lists from Progenesis and loaded to Proteome Discoverer (v2.1).
- Proteins were identified using Mascot (v2.5).

For Chip-seq analysis:
- Data was extracted from GSE58506 and GSE100292, aligned with GRCh37/hg19 and visualized with the Integrative Genomics Viewer (IGV) (v2.13.2).

For manuscripts utilizing custom algorithms or software that are central to the research but not yet described in published literature, software must be made available to editors and reviewers. We strongly encourage code deposition in a community repository (e.g. GitHub). See the Nature Portfolio guidelines for submitting code & software for further information.

Data

Policy information about availability of data

All manuscripts must include a data availability statement. This statement should provide the following information, where applicable:

- Accession codes, unique identifiers, or web links for publicly available datasets
- A description of any restrictions on data availability
- For clinical datasets or third party data, please ensure that the statement adheres to our policy

Data availability
The RNA-seq data discussed in this publication has been deposited in NCBI's Gene Expression Omnibus and are accessible through GEO Series accession number GSE175463 (https://www.ncbi.nlm.nih.gov/geo/query/acc.cgi?acc=GSE175463 ; token access number otstmcmibjurxmb).
The mass spectrometry data are available for revision via ProteomeXchange with identifier PXD026181 (Username: reviewer_pxd026181@ebi.ac.uk ; Password: Q142GpEX).
Publicly available datasets used in this study include PDX014088 (https://www.ebi.ac.uk/pride/archive/), GSE83332, GSE139505, GSE58506, GSE100292 (https://www.ncbi.nlm.nih.gov/geo/), GTex (https://gtexportal.org/ home/).
List of figures with associated raw data:
- Figure 1A (lncRNA screening)
- Figure 1B (GTex)
- Figure 1G and Supplementary 1A (LC-MS/MS)
- Figure 5 and Supplementary Figure 4; Figure 9B (RNA-seq).
- Figure 9A and Supplementary Table 3 (Mass Spectometry).
- Supplementary Figure 5E and F (ChipSeq).

Field-specific reporting

Please select the one below that is the best fit for your research. If you are not sure, read the appropriate sections before making your selection.

☒ Life sciences ☐ Behavioural & social sciences ☐ Ecological, evolutionary & environmental sciences

For a reference copy of the document with all sections, see nature.com/documents/nr-reporting-summary-flat.pdf

Life sciences study design

All studies must disclose on these points even when the disclosure is negative.

Sample size	No statistical method was used to predetermine the sample size. Sample sizes were chosen based on previous experience with similar type of experiments in our laboratory and published work by other groups.<br><br>For animal experiments, sample sizes followed Mead’s recommendations*. In particular, the accumulated n value (N) for a given comparison minus the number of groups or treatments (T) (for example, genotypes) was between 10 and 20, as recommended*.<br><br>* Festing, M. F. W., Overend, P., Gaines Das, R., Cortina Borja, M. & Berdoy, M. The design of animal experiments. Reducing the use of animals in research through better experimental design (Royal Society of Medicine Press, 2002).

Data exclusions

In the phosphoarray, technical replicates were excluded when non-quantifiable for technical reasons, but in all cases at least three replicates were quantified per protein. In in vivo studies, tumors (2 out of 48) or teratomas (1 out of 50) were excluded when they appeared as a significant outlier after performing an outlier Grubbs' test, also called the ESD method (extreme studentized deviate).
In RT-qPCR analysis, technical replicates were excluded when Tm values did not correspond to the expected gene amplification or when depicted as "Undetermined".

Replication

The experimental findings were reliably reproduced. The experiments shown in this manuscript were repeated independently at least twice obtaining similar results, unless specified.

Randomization

For in vitro experiments, cells were always plated at the same time and wells were randomly selected for different experimental groups or treatments.

Mice were randomly allocated to control and experimental groups before inoculating the cells.

Blinding

Investigators were not blinded to group allocation. Blinding was not possible as the same investigator performed cell seeding, treatments and end-point analysis. Automated quantitative methods were used to eliminate subjective interpretation of data.

Reporting for specific materials, systems and methods

We require information from authors about some types of materials, experimental systems and methods used in many studies. Here, indicate whether each material, system or method listed is relevant to your study. If you are not sure if a list item applies to your research, read the appropriate section before selecting a response.

Materials & experimental systems

n/a	Involved in the study	
☐	☒	Antibodies
☐	☒	Eukaryotic cell lines
☒	☐	Palaeontology and archaeology
☐	☒	Animals and other organisms
☐	☒	Human research participants
☒	☐	Clinical data
☐	☒	Dual use research of concern

Methods

n/a	Involved in the study	
☒	☐	ChIP-seq
☒	☐	Flow cytometry
☒	☐	MRI-based neuroimaging

Antibodies

Antibodies used

See Extended Data Table 7.

Validation

pTINCR antibody was validated for Western blot, immunofluorescence and immunohistochemistry using pTINCR-KO cells.
The expression patterns and/or subcellular localization of all the proteins analyzed by using comercial antibodies have been previously validated by the manufacturers and widely used in research. Methods of validation such as immunofluorescence and western blot and references have been published for all antibodies and are all present into manufacturer dedicated website of each indicated product.

HA-Tag, Ab9110 Abcam, PMID: 35217669, 35045281, 35118068
HA-Tag, H6908 Sigma, PMID: 31244224, 30356106, 30765691
GAPDH, AM4300 Thermo Fisher Scientific, PMID: 35847979, 35840569, 29479319
CDC42(B8), SC-8401 Santa Cruz, PMID: 30612298, 31358811, 31363101
LaminB1, PA5-19468 Thermo Fisher Scientific, PMID: 32528060, 32587248, 32931478
Tubulin, SC-32293 Santa Cruz, PMID: 35078997, 35215018, 3520581
Histone3, Ab18521 Abcam, PMID: 31822471, 32429384, 32555348
VDAC, SC-390996 SantaCruz, PMID: 32755582, 32848607, 34072974
ZO1, GTX108592 GeneTex, PMID: 31372515
b-Catenin, GTX633010 GeneTex, PMID: 32030757
E-Cadherin, 610182 BD, PMID: 31540219, 31535973, 31543815
SUMO1, 4930S Cell Signaling, PMID: 28569748, 28902364, 24385917
SUMO2/3, 4971S CellS ignaling, PMID: 29352108, 30348749, 29079793
HIS-Tag(D3I1O)XP®, 12698 Cell Signaling, PMID: 33658516, 33893293, 33020664
FLAG-Tag, F1804 Sigma, PMID: 35844805, 35860553, 35908039
INVOLUCRIN(cloneSY5), MS-126P1 Thermo Fisher Scientific, PMID: 31220456, 31564077, 30208113
p53(DO-1), SC-126 Santa Cruz, PMID: 36087186, 35732707, 35752616
B23, SC-5564 Santa Cruz, PMID: 23013792, 28981686, 33367824

Eukaryotic cell lines

Policy information about cell lines

Cell line source(s)	HEK293T, U2OS and A549 cell lines were obtained by ATCC. MCF7 cells were shared by the lab of Juan Angel Recio (VHIR, Barcelona, Spain). HaCaT cells were shared by the group of Manuel Serrano (IRB Barcelona, Spain). mESC V6.4 cell line was provided by Sagrario Ortega (Transgenic Mice Unit,CNIO, Madrid, Spain). cSCC-patient derived hSCC10 cell line was derived by Purificación Muñóz as described [48, 49].
Authentication	All cell lines were authenticated by genetic profiling using polymorphic short tandem repeat (STR) loci, except U2OS and mESC V6.4 cells that were not authenticated.
Mycoplasma contamination	Cell lines were routinely tested for mycoplasma contamination by PCR and confirmed negative.
Commonly misidentified lines (See ICLAC register)	No commonly mis-identified cell lines were used in this study.

Animals and other organisms

Policy information about studies involving animals; ARRIVE guidelines recommended for reporting animal research

Laboratory animals	We have used 8-week-old immunocompromised male NMRI (RjHan:NMRI) mice for teratoma assays and for generating PDXs.
Wild animals	We have not used any wild animal in our studies.
Field-collected samples	We have not used any field-collected samples in our studies.
Ethics oversight	Our studies comply with the European, Spanish and Catalan Regulations for the Protection of Vertebrate Animals used for Experimental and other Scientific Purposes (Directive 2010/63; Spanish BOE RD 53/2013; Catalan DOGC 214/1997). All studies were carried out in the "Lab Animal Service of Campus Vall d'Hebron (LAS-CVH)", registered and accredited at the Departament de Medi Ambient i Habitatge by Generalitat of Catalonia government with register number B9900062. The experiments performed for this manuscript were linked to a project approved by the Ethical Committee of Animal Experimentation of the Vall d'Hebron Campus, and the Commission of Animal Experimentation of the Department of Territory and Sustainability of the Generalitat of Catalonia under number 11156.

Note that full information on the approval of the study protocol must also be provided in the manuscript.

Human research participants

Policy information about studies involving human research participants

Population characteristics	We obtained biobanked cutaneous squamous cell carcinoma (cSCC) tissue samples from 51 cancer patients. 35 patients were males and 16 females, aged between 33 and 90 years.
Recruitment	All patient samples studied have the approval of the Ethics Committee for Research of the Vall d'Hebron University Hospital for molecular studies. Recruitment was not done specifically for our study.
Ethics oversight	Our studies have been performed in accordance with the Declaration of Helsinki. The protocol for human sample biobanking was approved by the Ethics Committee for Research of the Vall d'Hebron University Hospital (PR reseach project PR research project (AG) 191/2019)).

Note that full information on the approval of the study protocol must also be provided in the manuscript.

Dual use research of concern

Policy information about dual use research of concern

Hazards

Could the accidental, deliberate or reckless misuse of agents or technologies generated in the work, or the application of information presented in the manuscript, pose a threat to:

No	Yes	
☒	☐	Public health
☒	☐	National security
☒	☐	Crops and/or livestock
☒	☐	Ecosystems
☒	☐	Any other significant area

Experiments of concern

Does the work involve any of these experiments of concern:

No	Yes	
☒	☐	Demonstrate how to render a vaccine ineffective
☒	☐	Confer resistance to therapeutically useful antibiotics or antiviral agents
☒	☐	Enhance the virulence of a pathogen or render a nonpathogen virulent
☒	☐	Increase transmissibility of a pathogen
☒	☐	Alter the host range of a pathogen
☒	☐	Enable evasion of diagnostic/detection modalities
☒	☐	Enable the weaponization of a biological agent or toxin
☒	☐	Any other potentially harmful combination of experiments and agents
