# Supplementary material for: pTINCR microprotein promotes epithelial differentiation and suppresses tumor growth through CDC42 SUMOylation and activation
Source: Nat Commun. 2022 Nov 11;13:6840. doi: 10.1038/s41467-022-34529-6 (PMC9652315; doi:10.1038/s41467-022-34529-6)
Supplement: Supplementary file 4 — Source Data File [file 41467_2022_34529_MOESM4_ESM.zip › SOURCE DATA BLOTS.pptx]

## Slide 1
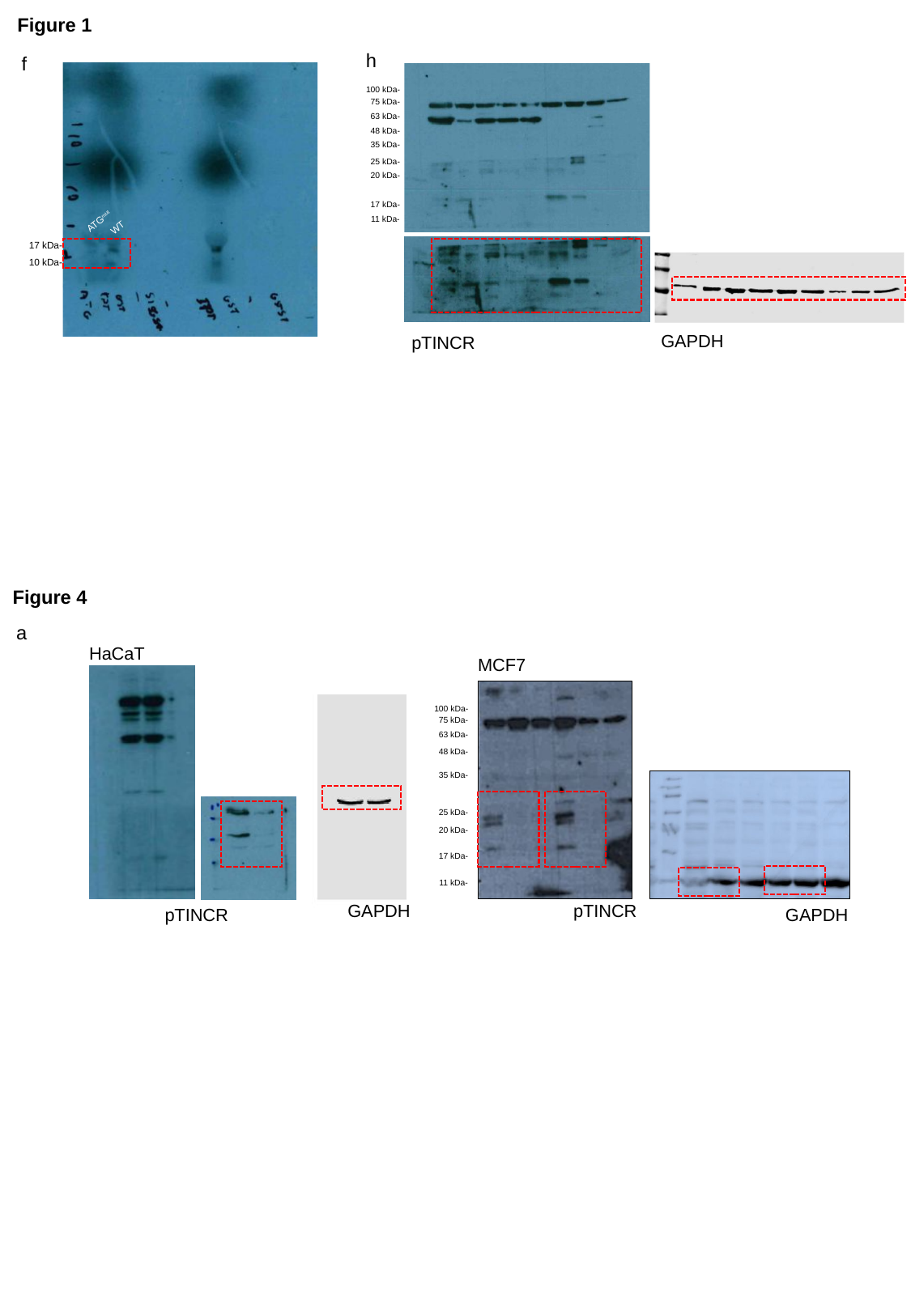

Figure 1
h
f
100 kDa-
75 kDa-
63 kDa-
48 kDa-
35 kDa-
25 kDa-
20 kDa-
17 kDa-
ATGmut
11 kDa-
WT
17 kDa-
10 kDa-
GAPDH
pTINCR
Figure 4
a
HaCaT
MCF7
a
100 kDa-
75 kDa-
63 kDa-
48 kDa-
35 kDa-
25 kDa-
20 kDa-
17 kDa-
11 kDa-
pTINCR
GAPDH
GAPDH
pTINCR

## Slide 2
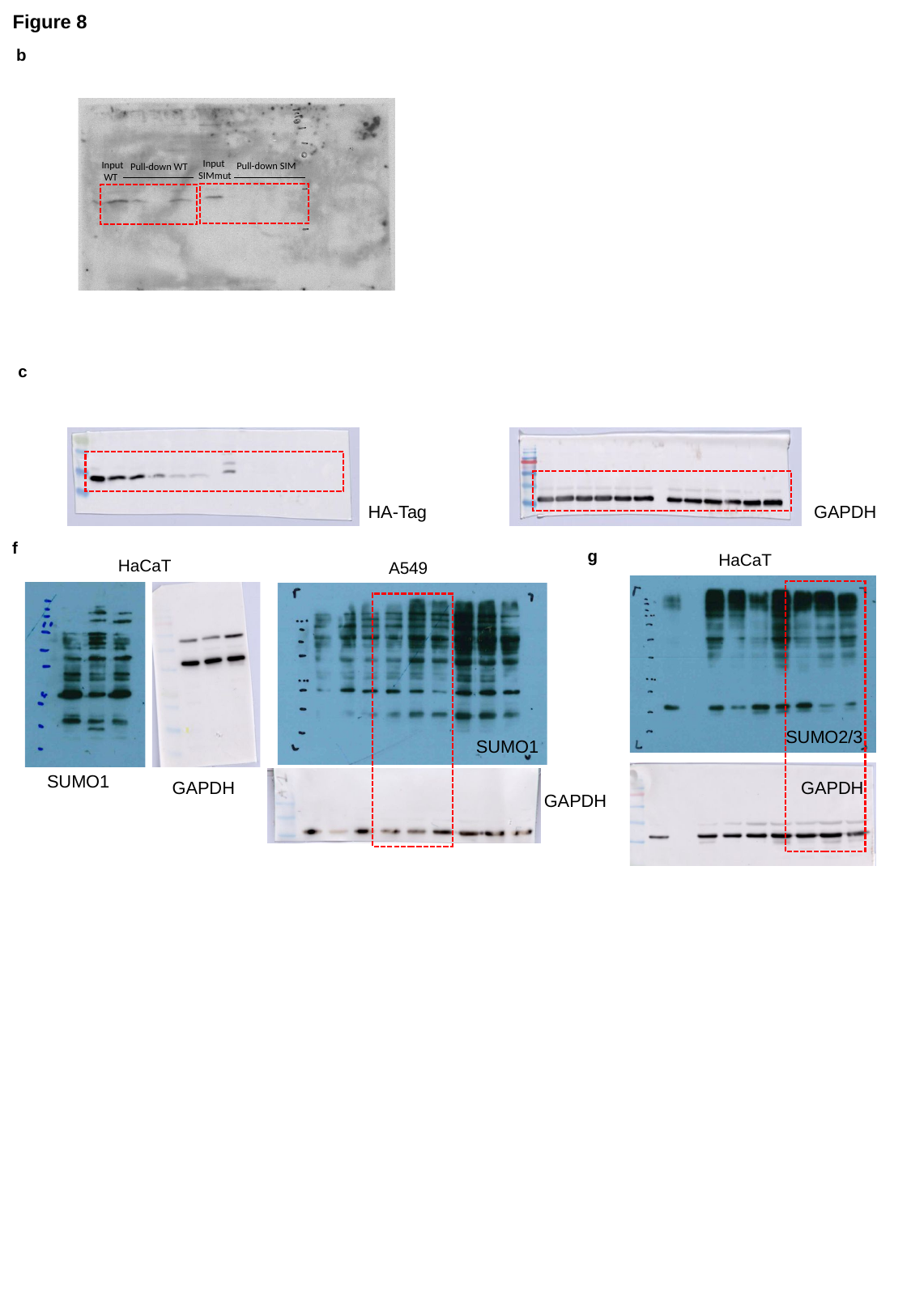

Figure 8
b
Input
 SIMmut
Input
 WT
Pull-down SIM
Pull-down WT
c
HA-Tag
GAPDH
f
g
HaCaT
HaCaT
A549
SUMO2/3
SUMO1
SUMO1
GAPDH
GAPDH
GAPDH

## Slide 3
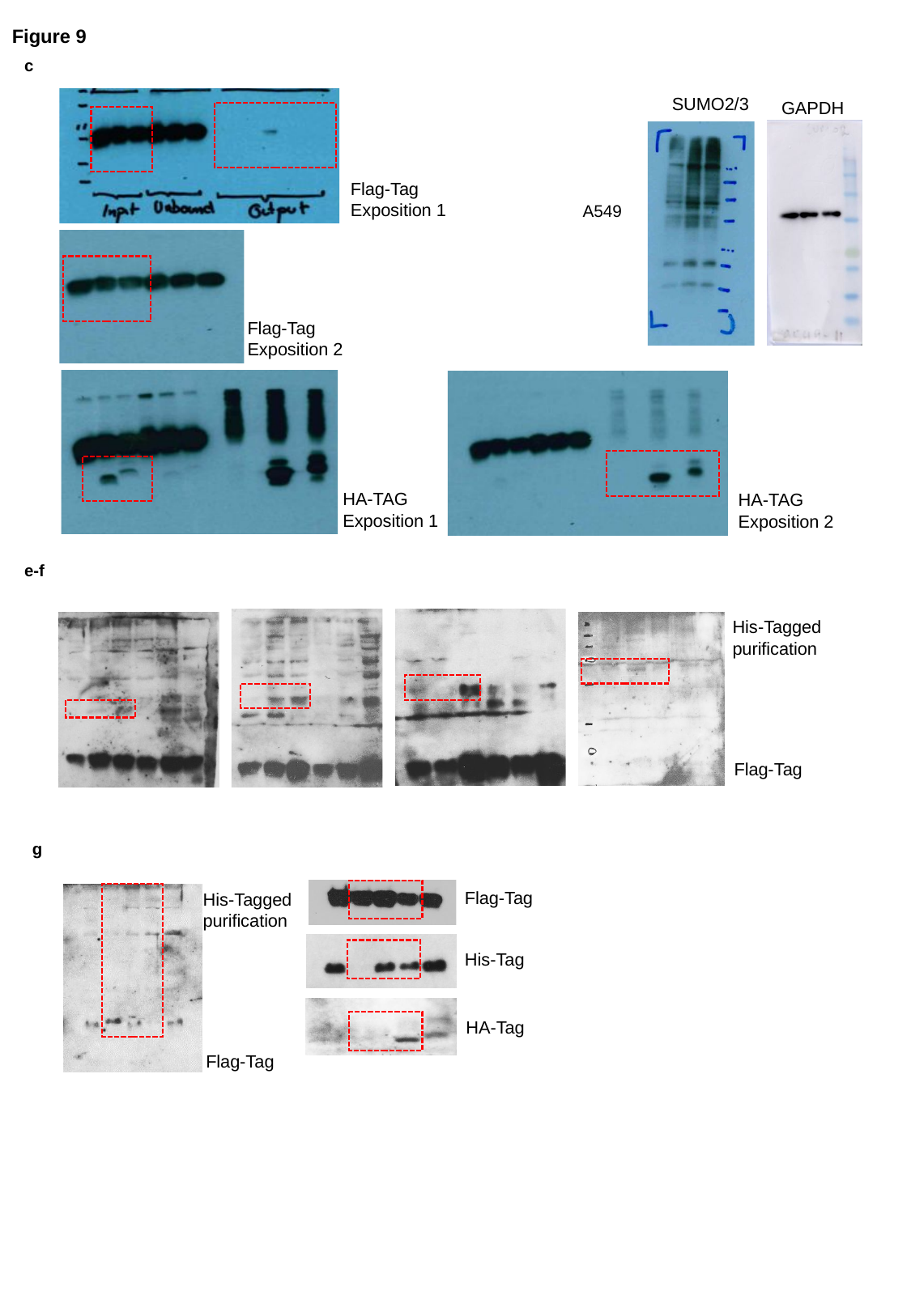

Figure 9
c
SUMO2/3
GAPDH
Flag-Tag
Exposition 1
A549
Flag-Tag
Exposition 2
HA-TAG
Exposition 1
HA-TAG
Exposition 2
e-f
His-Tagged purification
Flag-Tag
g
Flag-Tag
His-Tagged purification
His-Tag
HA-Tag
Flag-Tag

## Slide 4
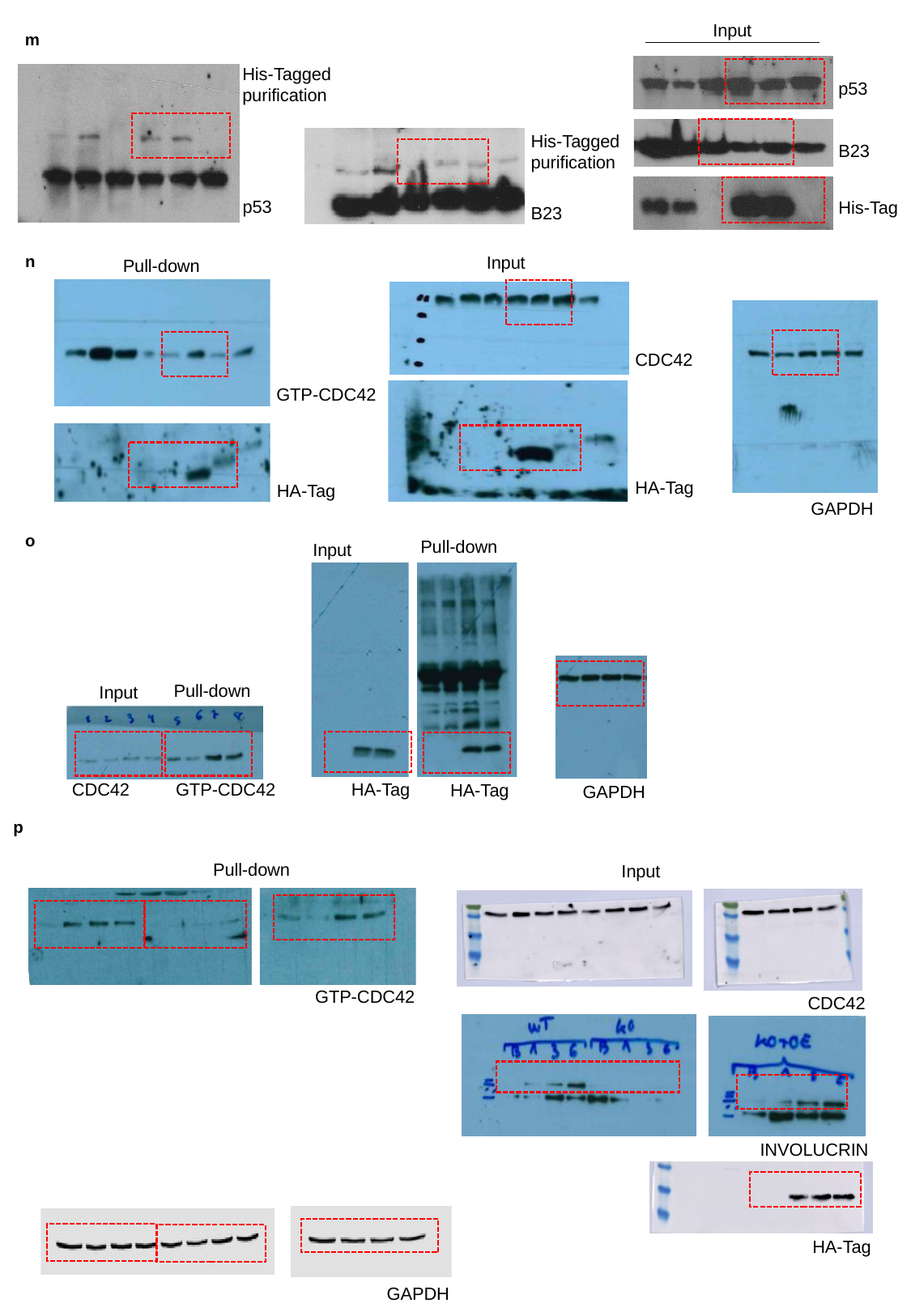

Input
m
His-Tagged purification
p53
His-Tagged purification
B23
p53
His-Tag
B23
n
Input
Pull-down
CDC42
GTP-CDC42
HA-Tag
HA-Tag
GAPDH
o
Pull-down
Input
Pull-down
Input
CDC42
GTP-CDC42
HA-Tag
HA-Tag
GAPDH
p
Pull-down
Input
GTP-CDC42
CDC42
INVOLUCRIN
HA-Tag
GAPDH
